# Genetic Diversity and Phylogeography of the Relict Tree Fern *Culcita macrocarpa*: Influence of Clonality and Breeding System on Genetic Variation

**DOI:** 10.3390/plants13121587

**Published:** 2024-06-07

**Authors:** Víctor N. Suárez-Santiago, Jim Provan, Ana Teresa Romero-García, Samira Ben-Menni Schuler

**Affiliations:** 1 Department of Botany, Faculty of Sciences, University of Granada, 18071 Granada, Spain; atromero@ugr.es; 2 Institute of Biological, Environmental and Rural Sciences, Aberystwyth University, Aberystwyth SY23 3DA, UK

**Keywords:** breeding system, clonality, *Culcita macrocarpa*, fern phylogeography, genetic diversity, glacial refugia, microsatellites, palaeotropical flora, plastid DNA, species distribution modeling

## Abstract

The tree fern *Culcita macrocarpa*, a threatened Iberian–Macaronesian endemism, represents the sole European species of the order Cyatheales. Considered a Tertiary relict of European Palaeotropical flora, its evolutionary history and genetic diversity, potentially influenced by presumed high clonal propagation, remain largely unknown. This study elucidates the phylogeographic history of *C. macrocarpa*, assessing the impact of vegetative reproduction on population dynamics and genetic variability. We provide genetic data from eight newly identified nuclear microsatellite loci and one plastid DNA region for 17 populations spanning the species’ range, together with species distribution modeling data. Microsatellites reveal pervasive clonality in *C. macrocarpa*, which has varied among populations. We assess the impact of clonality on genetic diversity and evaluate how estimates of intra-population genetic diversity indices and genetic structuring are affected by the chosen definition of “individual” (focusing exclusively on genetically distinct individuals, genets, as opposed to considering all independent clonal replicates, ramets). We identify two main population groups, one in the northern Iberian Peninsula and the other in the Macaronesian archipelagos and southern Iberian Peninsula. Within each group, we found relict populations (in the Azores and the Cantabrian Cornice) as well as recent originated populations. This population structure suggests colonization dynamics in which recent populations originated from one or a few genets of relict populations and became established through intra-gametophytic self-fertilization and vegetative expansion. DAPC analysis facilitated the identification of alleles that most significantly contributed to the observed population structure. The current Andalusian populations appear to have originated from colonization events from the Azores and the Cantabrian Cornice. Our findings suggest that *C. macrocarpa* persisted through the Last Glacial Maximum in two refugia: the Azores and the Cantabrian Cornice. Colonization into new areas occurred presumably from these refuges, generating two large population groups with structured genetic diversity. This study underscores the significance of clonality in establishing new populations and shaping genetic structure.

## 1. Introduction

Although ferns are the second most diverse group of vascular plants after angiosperms (*ca*. 11,000 species; [1]), there is a comparative lack of data on population genetics and phylogeography for this important group [2]. Numerous ferns are considered lineages of Tertiary origin, especially those from the Mediterranean region [3,4], which constituted the main component of the herbaceous layer of the European lauroid forest [5,6,7] and survived the Quaternary glaciations in well-characterized glacial refugia, such as the Iberian Peninsula, Italy, the Balkans, and Greece [8].

Ferns, with their high capability for spore-mediated dispersal [9], usually exhibit strong population connectivity and reduced genetic structure [10]. However, the limited availability and disjunct distribution of suitable habitats with high moisture and warm temperatures, especially for southern European Tertiary ferns, may result in pronounced genetic structuring (e.g., [11,12,13,14,15,16,17,18,19,20]). In ferns, as in many other non-seed plants, fertilization is a post-dispersal process (occurring on the gametophyte from spores already dispersed), and consequently the probability of colonizing a new habitat as well as the genetic composition of the new population will depend on the breeding system of the species. The colonization process will be less probable for ferns with outcrossing and inter-gametophytic selfing, and the resulting populations will be genetically more diverse. By contrast, those with intra-gametophytic selfing will be able to establish a new population even from a single spore, but will result in populations with very low diversity [21,22]. Moreover, many ferns also have the ability to propagate vegetatively, further shaping levels and patterns of within- and between-population diversity. Clonal reproduction will tend to decrease in within-population genetic diversity due to a lack of meiosis and recombination or because interclonal competition can lead to the elimination of less adapted clones, even to the extent of forming monoclonal populations [23]. However, clonality tends to increase heterozygosity by the accumulation of mutation and divergence between alleles [24,25]. Population differentiation, estimated as the *F*_ST_ value, is drastically reduced when reproduction tends toward strict clonality because genetic diversity is maintained within clonal lineages. However, even very limited proportions of sexual reproduction make the effect of clonality on population differentiation negligible [24].

**Figure 1:**
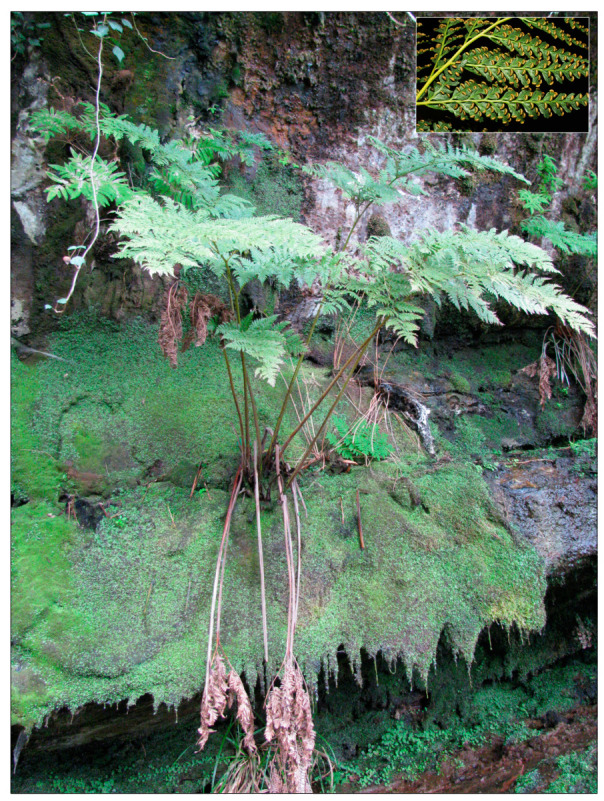
*Culcita macrocarpa*. Individual from the Almoraima population in Cádiz province (Andalusia, Spain), exhibiting the subarborescent habit. Inset (upper right), detail of a frond with sori. Photos: Gabriel Blanca.

In the present study, we focus on *Culcita macrocarpa* C. Presl (Culcitaceae; Figure 1), a diploid tree fern considered to be a relict from Palaeotropical flora [3]. It has a subarborescent-creeping habit with a thick rhizome covered with reddish filiform scales. The fronds can exceed 2 m in length, and on the margins of these the reniform sori develop ([26]; Figure 1). *Culcita macrocarpa* is an Iberian–Macaronesian endemic species (Andalusia, the Cantabrian Cornice, the Azores, the Canary Islands, and Madeira), and the only European representative of the genus, since the other known species [*C. coniifolia* (Hook.) Maxon] has an exclusively American distribution. According to other authors [27,28], the divergence between the lineages of the two species occurred about 20 million years ago. *Culcita macrocarpa* is restricted to shady sites with mild temperatures throughout the year and high humidity and soil moisture, as in valleys near the coast, usually at altitudes below 300 m, or in higher areas associated with fog belts (even above 1000 m), especially in the Azores. As many of these habitats where *C. macrocarpa* is distributed are under threat, and given its disjunct distribution, the species is currently protected under the Bern Convention and the Habitats Directive [29,30]. In addition to being an endangered species in the Red List of Spanish Vascular Flora, it is also included in the Spanish List of Wild Species under Special Protection [31,32]. Also, this species is also considered critically endangered in the Red List of the Vascular Flora of Mainland Portugal [33].

Reproductively, culture experiments have shown that gametophytes of *C. macrocarpa* are initially male and later become hermaphrodite, and that they do not produce antheridiogens [34,35], a scenario that seems to favor intra-gametophytic selfing [36,37]. A study was performed on the genetic variation in six populations from Galicia (north-western Iberian Peninsula) using 13 enzymatic systems [38], seeking to assess the concordance between genotypic frequencies and the breeding system. However, no insights were gained into the reproductive system of the species, since only a single multilocus genotype was found across all individuals and populations, although evidence was found for the intense clonal reproduction of the species [38]. *Culcita macrocarpa* has a creeping rhizome that can exceed one meter in length, bifurcating and giving rise to new shoot apices that form adventitious roots, each shoot apex constituting a separate clone or ramet [35,38,39]. The lack of observed genetic variation was attributed by the authors to genetic drift associated with the reduction in the number of populations during the last ice age, and subsequent founder effects during Holocene expansion [38].

The evolutionary history of *C. macrocarpa* remains unknown. Furthermore, as noted above, molecular genetic approaches have not as yet been successfully used to evaluate the effect of clonality on its intraspecific genetic structure. To quantify the genetic variation and its distribution within and between populations, and to identify suitable areas for the species in the past and the future, we used nuclear microsatellite loci, one plastid marker, and species distribution modeling (SDM) with a double aim. Firstly, we sought to infer the species’ phylogeographic history. The biogeographic pattern of the species, typical of a relict taxon, makes *C. macrocarpa* a good system to assess the impact of both pre-Pleistocene and Quaternary geological and climatic events on population processes that generate genetic structure, and present insights into how ongoing and future climate change may shape evolutionary patterns. Furthermore, phylogeographic data from ancient taxa, such as *C. macrocarpa*, may also provide insights into the demise of the Palaeotropical flora of Europe. Secondly, we sought to estimate the importance of sexual and vegetative reproduction on population composition and to evaluate the effect of clonality on intraspecific genetic structure.

## 2. Materials and Methods

### 2.1. Plant Material

**Figure 2:**
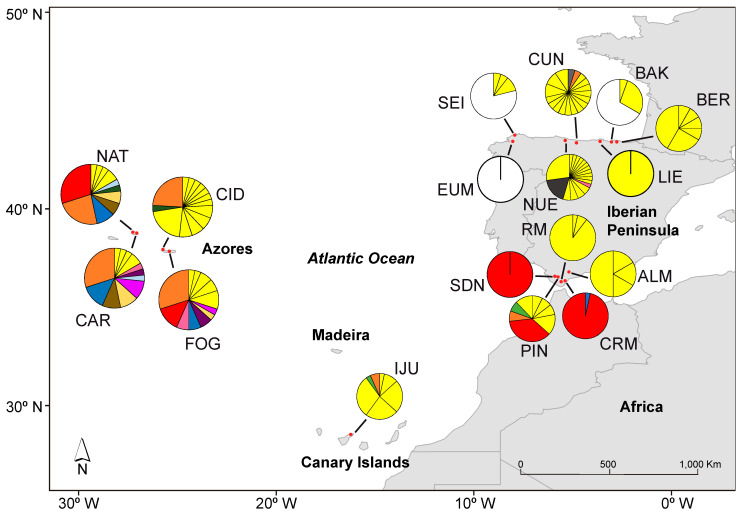
Map of the *Culcita macrocarpa* sampling locations. Frequency of each multilocus lineage (MLL) based on microsatellite data is represented as pie charts per location. Colors represent the different MLLs that are shared among sites, and yellow represents private MLLs that only appear in one site. See Table 1 for the full name of locations. Note that the pie chart size is the same for all locations and does not represent the number of individuals.

A total of 443 individuals of *C. macrocarpa* was sampled from 17 populations in 4 geographical regions across its distribution range: Andalusia, the Azores, the Canary Islands, and the Cantabrian Cornice. In Madeira, where the species is reportedly also present, we could not find any population. The number of populations per region was between one and five, and the number of sampled individuals per population varied from 6 to 30 (Table 1; Figure 2).

### 2.2. DNA Extraction, Microsatellite Genotyping, and Plastid DNA Sequencing

Total genomic DNA of the 443 individuals was extracted from silica-dried fronds following a modified low-salt CTAB extraction protocol [40]. We developed eight microsatellite loci, which were used to genotype all individuals (see Table S1 for microsatellite characteristics). Genotyping was performed on an ABI PRISM^®^ 3100-Avant Genetic Analyzer (Applied Biosystems, Foster City, CA, USA). Alleles were scored using GENEMARKER v1.85 (SoftGenetics, State College, PA, USA).

For plastid DNA (ptDNA) analysis, a subsample of 82 individuals (3–5 per population) was used. The plastid marker used was the intergenic spacer *rps4-trnL*, the only polymorphic region of the 13 tested (introns in *rpl16*, *rps16*, *trnG*^UCC^, *trnL*, and *ycf3*, and the intergenic spacers *rpl32-trnL*, *rps4-trnL*, *rps16-matK*, *trnD-psbM*, *trnD-rpoB*, *trnH-psbA*, *trnL-trnF*, and *trnS*^GGA^*-trnG*^UCC^). All primer pairs used for PCR amplification are shown in Table S2. PCR reactions were performed in 25 µL reactions containing 50 ng of genomic DNA, 1.25 µM of each primer, 2 mM MgCl_2_, 1.5 mM dNTPs, 2.5 µL Biotools 10× Reaction Buffer, and 1.5 units of Biotools DNA polymerase (Biotools B&M Laboratories S.A., Madrid, Spain). Cycling parameters consisted of 5 min of denaturing at 94 °C; followed by 35 cycles of 94 °C for 1 min, 50 °C for 1 min, and 72 °C for 1 min; and a final extension at 72 °C for 10 min. Sequencing was performed on an ABI PRISM^®^ 3100-Avant Genetic Analyzer (Applied Biosystems, Foster City, CA, USA). The resulting sequences were aligned by eye using the alignment editor BIOEDIT v7.0.5.3 [41].

### 2.3. Clonality and Genetic Diversity

#### 2.3.1. Microsatellites

To infer the clonal identity of the sampling units (all individuals sampled), and to discriminate between genetically distinct individuals resulting from different zygotes (genets) and independent clonal replicates genetically identical to the parent plant (ramets), we firstly tested the resolutive power of the eight microsatellite markers by estimating the genotype accumulation curve using the function *genotype_curve* from the R package POPPR v2.8.3 [42]. This function samples random subsets of loci by the use of a Monte Carlo procedure, and examines the robustness of the inferred clonal memberships. Then, we used MLGsim v2.0 [43] to calculate the probability that repeated multilocus genotypes (MLGs) originated from different sexual reproduction events (*p*_sex_; being genetically distinct individuals, different genets). This was based on the observed allele frequencies and the sample size of the data set, while taking into account departures from the Hardy–Weinberg equilibrium (HWE) when estimating genotypic probabilities (*p*_gen_ (*F*_IS_), for a more conservative estimate of *p*_sex_; [44]). The significance values of *p*_sex_ were determined by comparison with the distribution of 1000 simulated *p*_sex_ values. Finally, to define the clonal lineages or multiple locus lineages (MLLs; i.e., different MLGs belonging to a different or the same clone), we analyzed the distribution of the frequencies of genetic distances between pairs of MLGs, with the function *mlg.filter* and using Bruvo distances on POPPR. The genetic threshold distance under which two MLGs were considered the same MLL was estimated using the farthest-neighbor method.

The clonality descriptors were calculated with the function *poppr* on POPPR as follows. Firstly, to characterize the clonal richness, we determined the number of MLLs, the number of expected MLLs (eMLLs), and the clonal richness (R) corrected for the sampling size. Secondly, to characterize the genotype diversity, we calculated the Simpson’s index (lambda; corrected for sampling size) and the clonal evenness index (E.5), which shows how equally each MLL is represented. Finally, we calculated the standardized association index (*r*_d_; [45]) to test the predominant reproductive model (sexual, where linkage among loci is not expected, vs. clonal, where significant disequilibrium is expected due to linkage among loci). This latter index was also estimated correcting for clones and then using only one individual per MLL, in order to test the effect of partial clonality. The significance of *r*_d_ was tested with a permutation test (10,000 permutations). All descriptors were estimated both at the population and geographical region levels.

To calculate genetic diversity descriptors, we used all sampling units (including ramets), following the recommendation of [46], because this way, the real genetic structure of *C. macrocarpa* populations is more accurately represented. Thus, we calculated: the number of alleles (*A*) and allelic richness (*Ar*), rarefacting to the smallest sample size, using the R package HIERFSTAT v0.04-30 [47] with the functions *allele.count* and *allelic.richness*, respectively; the observed and expected heterozygosity (*H*_O_ and *H*_E_, respectively), and fixation index *F*_IS_ and HWE, using GENODIVE v3.0 [48]. Furthermore, to investigate how the definition of the individual that we used (ramet-based vs. genet-based definitions) influences the estimates of intra-population genetic diversity indices (as recommended by [49]), we also calculated genetic descriptors (*Ar*, *H*_O_, *H*_E_, and *F*_IS_) using only one individual per MLL per population (only genets). Student’s *t* tests were performed to explore significant differences between data sets (including vs. excluding clones). All genetic descriptors were calculated at the population and geographical region levels.

#### 2.3.2. ptDNA

Genetic diversity was assessed by the number of haplotypes (*ha*), haplotype diversity (*Hd*), and nucleotide diversity (*π*) calculated using ARLEQUIN v3.5.2.2 [50]. All diversity indices were calculated at the population and geographical region levels.

### 2.4. Genetic Structure and Phylogeography

Standard and hierarchical analyses of molecular variance (AMOVA; [51]) were used to test for partitioning of genetic variability within samples, within and between populations, and between the four geographical regions. For microsatellites, these analyses were made with all sampling units (including ramets) and with only one individual per MLL per population (genets), using the function *poppr.amova* on POPPR and the function *randtest*, running 1000 replicates, to test for significance. For ptDNA, we used the program ARLEQUIN, and the significance was tested with 10,000 permutations. In addition, one more AMOVA analysis was carried out for ptDNA considering the geographical distribution of the haplotypes and the result of the network analysis (see the Results Section). Thus, we included a fourth level of population grouping called supra-regional grouping. Two supra-regional groups were considered: South (Andalusia, the Azores, and the Canary Islands) and the Cantabrian Cornice.

Population genetic structure was analyzed using different approaches with our microsatellite data. First, pairwise *F*_ST_ values were calculated, both with all sampling units and with only one individual per MLL per population, between populations using GENODIVE; the significance of *F*_ST_ was tested by a permutation test with 10,000 permutations. We compared the values found with and without clones using the Spearman’s correlation coefficient. Second, the Bayesian algorithm implemented in STRUCTURE v2.3.4 [52] was used to evaluate the number of genetic clusters (*K*) both with all sampling units and with clone correction. The number of clusters tested ranged from one to 18, with 10 replicates per *K*, using the no-admixture model and independent allele frequencies. The burn-in period and Markov Chain Monte Carlo (MCMC) iterations were set to 50,000 and 10^6^, respectively. The optimal number of clusters was estimated with the online tool STRUCTURESELECTOR [53]. We identified the uppermost hierarchical level of genetic structure using the delta *K*-method (Δ*K*; [54]). To explore other levels of genetic partitioning, we used the four independent estimators proposed by [55] (MedMedK, MedMeaK, MaxMedK, and MaxMeaK), considering a membership coefficient threshold of 0.5. To align and visualize the STRUCTURE output across the 10 replicates, we used the online tool CLUMPAK v1.1 [56]. Third, the genetic structure was also assessed using a model-free multivariate statistics-based clustering method, a discriminant analysis of principal components (DAPC) on R package ADEGENET v2.1.10 [57] using all sampling units. The function *xvalDapc* from ADEGENET was used to select by cross-validation the correct number of principal components with 1000 replicates using a training set of 90% of the data. The number of principal components was chosen based on the criteria that it had to produce the highest average percentage of successful reassignment and lowest root mean squared error [57].

The evolutionary relationships and geographical distribution of ptDNA haplotypes were explored by reconstructing a haplotype network following the statistical parsimony method [58] as implemented in TCS v1.21 [59].

### 2.5. Gene Flow Using Microsatellite Data

We tested the connectivity among populations by estimating the migration rates among them, consistently with all sampling units. Thus, to determine whether recent (over two to three generations) gene flow had occurred between the populations, we estimated the migration rates (*m*) between all individual populations using a Bayesian assignment test with the software BAYESASS v1.3 [60]. As program settings, the default values were used (MCMC iterations, 3 × 10^6^; length of the burn-in, 999,999; sampling frequency, 2000; delta value, 0.15). Isolation by distance (IBD) was tested for the 17 populations using the regression of pairwise *F*_ST_ distances [determined with GENODIVE using them transformed as *F*_ST_/(1 − *F*_ST_)] and logarithms of geographical distances between populations, by applying a Mantel test in GENODIVE.

### 2.6. Species Distribution Modeling

Potential refuge and future distribution areas for *C. macrocarpa* were determined by performing species distribution modeling (SDM). This analysis requires the presence occurrence data of the species studied and environmental variables. For environmental data, we used 19 BIOCLIM variables at a resolution of 2.5 arc minutes (ca. 5 km) representing different time periods during past, present, and future climatic conditions. Past and current climate data were available from the WorldClim database (www.worldclim.org; [61]) and included data for the current-day period (1950–2000), the Last Glacial Maximum (LGM; *c*. 21 ka) simulated by the CCSM model (the Community Climate System Model), and for the Last Interglacial period (LIG; *c*. 120 ka). We obtained predictions for future climatic conditions in the year 2080 for the IPCC climate scenario with the most impact: RCP8.5 [62] available through the CCAFS Climate portal (www.ccafs-climate.org). Soil data were derived from SoilGrids.org [63] but were not used with past climatic conditions in the LGM because of the lack of such maps. Highly correlated variables (Pearson’s R ≥ 0.8) were reduced to eight uncorrelated variables (Table S3) used as predictors to calibrate the distribution models. Species-occurrence data are a collection of references in databases (the Global Biodiversity Information Facility data portal (http://www.gbif.org/; accessed on 15 July 2017), the Biodiversity databank of the Canary Islands (http://www.biodiversidadcanarias.es/atlantis/common/index.jsf; accessed on 16 June 2013), the Azores Biodiversity databank (http://www.atlantis.angra.uac.pt/atlantis/common/index.jsf; accessed 1 February 2014), the literature [64,65,66,67], plus our own field records. A total of 379 presence records were finally compiled (Figure S1). To perform the SDM, we applied maximum entropy modeling implemented in the software package MAXENT v3.4.1 [68]. Models were generated using a cross-validation of 5 replicate runs. Model performance was assessed based on the area under the receiver operating characteristic curve (AUC). The contribution of each predictor variable in the model was analyzed by the permutation importance and percent contribution coefficients (Table S3). A final reduced model including the most important variables [69], i.e., the mean diurnal range and minimum temperature of coldest month, was finally computed.

## 3. Results

### 3.1. Clonality and Genetic Diversity

#### Microsatellites

A total of 120 different multilocus genotypes (MLGs) were detected among the 443 sampling units. The genotype accumulation curve showed that our eight microsatellite loci had strong power to discriminate between the MLGs of *C. macrocarpa*, since with seven loci, almost 100% of the MLGs were resolved (Figure S2). Only for one repeated MLG could we not rule out an independent origin by sexual reproduction (*p*_sex_ = 0.96, *p* = 0.115). This MLG was shared between two sampling units of different populations, CAR (the Azores) and NUE (the Cantabrian Cornice), and therefore these and all the sampling units of different populations with shared MLGs were maintained in the data set when clone correction was applied. The genetic threshold distance under which two MLGs were considered to belong to the same multilocus lineage (MLL) was 0.0391 (Figure S3). After the MLGs were collapsed into MLLs, the total number of MLLs was 104 distributed among 130 individuals (genetically distinct individuals; genets) across the populations, with different numbers of clones between populations and geographical regions (Figure 2 and Figure S4A,B; Table 2). Between 1 and 23 MLLs were detected across all the populations. Thirteen MLLs were shared among populations, of which the most frequent showed a differential distribution between Andalusia, the Azores, and the Cantabrian Cornice (Figure 2, MLLs: red, orange, and white and black). The rest of the MLLs were exclusive to the populations (Figure 2; Table 2). The Cantabrian Cornice was the region that retained the highest number of MLLs (53) and clonal richness (R = 0.31), although this was not evenly distributed among the populations. Many Cantabrian populations had very low numbers of MLLs (two were even monoclonal, i.e., EUM and LIE), while CUN (23 MLG; R = 0.759) and NUE (18 MLG; R = 0.586) harbored the highest values of all populations sampled. On the contrary, in the Azores, despite having fewer MLLs (35, R = 0.29), their populations harbored relatively high and similar levels of clonal richness (Table 2), and the expected MLL (eMLL; Table 2) values were even higher than those of the Cantabrian Cornice. The differential prevalence of clones between the Azores populations and between those of the Cantabrian Cornice is reflected in the regional evenness index (Table 2; Figure S4A,B). Although the CUN and NUE populations showed a proportionate distribution of clones and therefore had a high evenness value, the dominance of a few clones in other populations (except BER) made the Cantabrian Cornice the region with the lowest value for evenness. The Azores, meanwhile, was the region with the highest proportionality in the distribution of clones (excluding the Canary Islands) and also with the greatest clonal diversity (lambda; Table 2). Conversely, Andalusia was the region with lowest values of clonal richness and genotype diversity, since the populations showed few MLLs and only some were dominant (Figure 2 and Figure S4A,B; Table 2). In the Canary Islands, although the only known population presented moderate to low values of clonal richness, the genotype diversity indexes were close to those found in the Azores and the most diverse populations of the Cantabrian Cornice (Table 2). All the populations for which the association index could be calculated, except FOG, NAT, and IJU, and geographical regions, except the Canary Islands, showed a significant linkage disequilibrium when all sampling units were included. When only one individual was considered per MLL per population, only PIN at the population level, and Andalusia and the Cantabrian Cornice at the regional level, presented significant disequilibrium (Table 2).

In total, 37 alleles were detected from the eight loci surveyed. Between two and five alleles per locus were found across all the populations (Table 3). Allelic richness and the expected heterozygosity (*H*_E_) significantly changed when only one individual per MLL per population was considered (*t* = 3.05; *p* < 0.05 and *t* = −2.69; *p* < 0.05 respectively), but the observed heterozygosity (*H*_O_) and the fixation index (*F*_IS_) did not change (*t* = −1.41; *p* < 0.17 and *t* = −1.04; *p* < 0.31, respectively). All populations, except ALM, deviated from HWE when all individuals were included, resulting in significantly negative values of *F*_IS_ in the populations CRM, RM, SDN, IJU, CUN, and LIE. When only one individual per MLL per population was considered, the *F*_IS_ values remained negative for these populations (except for SDN and LIE with only one MLL each) (Table 3). The negative values of *F*_IS_ were determined mainly by the locus *CM-AT19*, which showed fixed heterozygosity (for only two alleles) in almost all populations, and the high number of monomorphic loci in the different populations (Table S4). After *CM-AT19* was excluded, only the populations RM, IJU, and CUN registered negative *F*_IS_ values (significant only in RM). With respect to the regions, the *F*_IS_ values showed a significant excess of homozygotes (except the Canary Islands, with only one population), both including all sampling units and only one individual per MLL per population. At the population level, those of the Azores and Cantabrian Cornice (especially BER, NUE, and CUN) showed the highest diversity values, versus the lowest in Andalusia; however, at the regional level, Andalusia had diversity levels similar to those of the other regions (Table 3).

When considering the prevalence of vegetative propagation in the populations (clonal richness, R; Figure S5), on the one hand, we found an upward trend in the values of allelic richness (*Ar*) and the expected heterozygosity (*H*_E_) with decreasing clonality (higher R). However, no clear effect of clonality on observed heterozygosity (*H*_O_) was discerned, and populations with higher clonal prevalence in general had *F*_IS_ values farther from zero. On the other hand, clonality appeared to influence the extent of the differences between the estimates for the diversity indices when applying the different definitions of “individual” (considering clonal replicates, ramets, vs. including only genetically distinct individuals, genets). Thus, with the increase in clonality, the differences for the estimates of *H*_O_, *H*_E_, and *F*_IS_ increased, while for *Ar* the differences were greater the less clonal (greater R) the populations were (Figure S5). In the case of *F*_IS_, the differences were greater because, when we considered only the genets, the estimated values of the most clonal populations trended to zero.

Plastid DNA sequence alignment included 82 sequences in total, with 235 base pairs (bp) in length, and included two variable positions. The total number of haplotypes found was four. The results for the diversity indices are shown in Table 3. At the population level, the mean diversity values for the ptDNA were *Hd* = 0.52 and *π* = 0.0023, with 12 populations showing null diversity values and the highest value in NUE (*Hd* = 0.7, *π* = 0.0034; the Cantabrian Cornice). At the regional level, the most diverse regions were the Cantabrian Cornice and Andalusia (Table 3). Most of the populations showed only one haplotype.

### 3.2. Genetic Structure and Phylogeography

AMOVA analyses showed that, when all sampling units are included, the highest proportion of diversity lies in the interpopulation component (64.74%, *F* = 0.647), or between regions when the regional component is considered (37.97%, *F* = 0.379; Table 4). When we included only one individual per MLL per population, interpopulation or interregional variation decreased significantly, although values remained significantly high, and the highest proportion of variation resided within individuals.

Pairwise *F*_ST_ values with and without clonal individuals (excluding monoclonal populations, with only one individual after clone correction) showed a significant correlation (*r* = 0.945; *p* = 0.0001); lower and less paired significant differences were found when clones were excluded (Table S5). Significant differences arose between most comparisons when all sampling units were used. At the intra-regional level, Andalusia and the Cantabrian Cornice presented high *F*_ST_ values among several of their populations. Thus, in Andalusia, the ALM and RM were sharply differentiated from each other and from the population group CRM-SDN-PIN. In the Cantabrian Cornice, the populations or population groups EUM-SEI-BAK/NUE-CUN/BER/LIE showed strong differentiation. The populations from the Azores registered low *F*_ST_ values between them. At the inter-regional level, most comparisons markedly differed, this being less notable between the Andalusian populations CRM-PIN-SDN and those of the Azores. The *F*_ST_ values proved relatively low in the comparisons in which CUN was involved. The Canary Island population (IJU) was strongly differentiated from the rest, except with respect to PIN (Andalusia), the populations from the Azores, and CUN.

**Figure 3:**
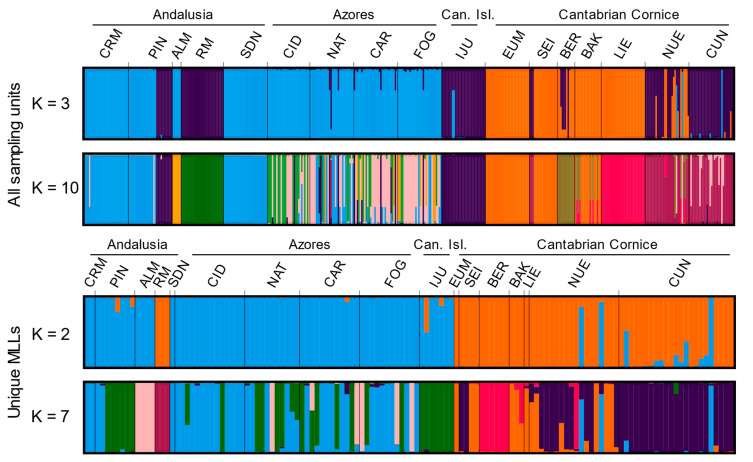
Estimated genetic structure based on microsatellite data using the Bayesian approach implemented in STRUCTURE v2.3.4. Histograms of individual assignments to clusters show the two most probable structures, *K* = 3 and *K* = 11, for all sampling units (ramet-based analyses), and *K* = 2 and *K* = 7 for analyses including only one individual per multilocus lineage per population (MLLs; genet-based analyses). The colors represent each of the genetic clusters identified. See Table 1 for full name of locations.

The results of STRUCTURE with and without clones are notably consistent with each other (Figure S6); although without clones, the structure is soon lost from *K* = 8, and the optimum number of selected clusters decreases from *K* = 3 and *K* = 10 (according to the Δ*K* and Puechmaille method, respectively) with clones up to *K* = 2 and *K* = 7 without clones. When *K* = 2 (optimal *K* without clones; Figure S6), one cluster was formed by the populations from Andalusia (minus RM), the Azores, and the Canary Islands, and another comprising the populations of the Cantabrian Cornice and RM (Andalusia); although NUE and CUN (the Cantabrian Cornice) had individuals more clearly defined as belonging to the first cluster (Figure 3). When *K* = 3 (optimal *K* with clones; Figure S6), a third cluster related the populations of RM, PIN (11 individuals of 30), IJU, several individuals of SEI and BER, NUE (18 individuals of 30), and CUN. Regarding the possibility of substructure (according to the Puechmaille method), for *K* = 10, the resulting clusters largely reflect the relationships found according to the pairwise *F*_ST_ values (i.e., ALM/CRM-PIN-SDN/RM/IJU-PIN/BER/EUM-SEI-BAK/LIE/NUE-CUN). The Azores populations appeared differentiated from the rest, although with a great mix between those of other clusters. Thus, most of the individuals from the Azores formed two clusters (pink and green in Figure 3), and many other individuals were better explained as belonging to clusters typical of other regions. The Andalusian population PIN showed a clear internal structure corresponding exactly to two intra-population nuclei (Figure 3).

**Figure 4:**
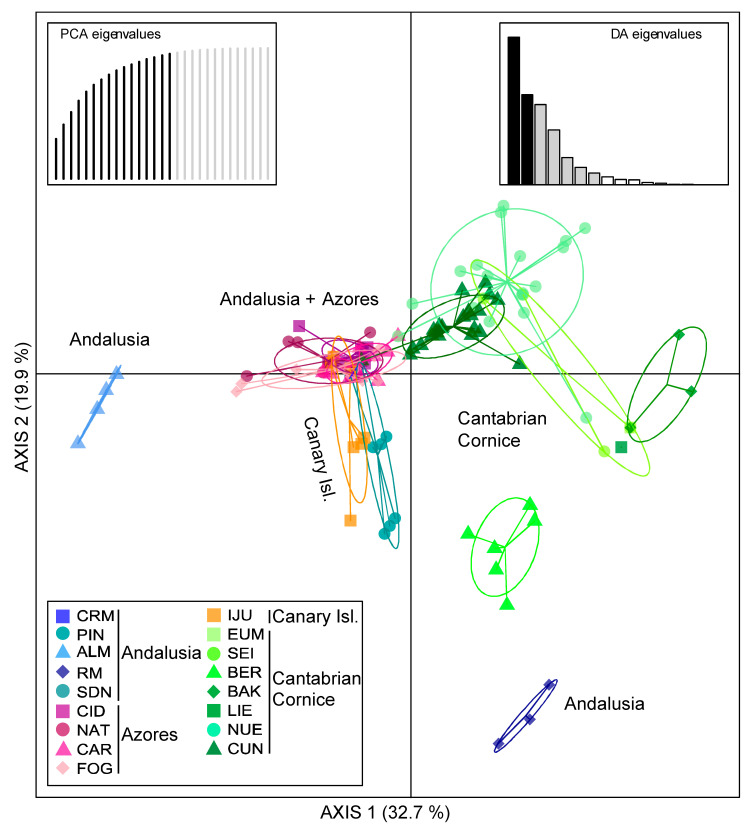
Result of the discriminant analysis of principal components (DAPC) using microsatellites. Scatterplot showing the first and second principal components. See Table 1 for full name of locations.

The DAPC analysis defined the relationships between the populations studied that closely reproduced the substructure that resulted in STRUCTURE, and the relationships identified with the pairwise *F*_ST_ values (Figure 4). The analysis suggested via the cross-validation that 16 principal components explained 95.6% of the variance in the original data. The resulting eigenvalues advised the inclusion of seven discriminant functions. Discriminant Function 1 explained 32.7% of the genetic variance, showing the differentiation between: the ALM population (Andalusia); the set of populations of the Azores, the rest of Andalusia (except RM), and the Canary Islands; the populations of the Cantabrian Cornice CUN, NUE, and BER, together with the Andalusian RM; and finally, the rest of the Cantabrian populations. Discriminant Function 2 explained 19.9% of the variance and differentiated the populations IJU (the Canary Islands), PIN (only individuals of an intra-populational nucleus) and RM (Andalusia), and BER (the Cantabrian Cornice) from the rest of the populations. The DAPC variable loadings included in the analysis revealed that loci *CM35*, *AT45m1*, and *AT9* (for Discriminant Function 1), and *CM1A* and *AT30* (for Discriminant Function 2), provided the highest resolution values for the individual assignment (Figure S7). Discriminant Function 3, which explained 17.8% of the genetic variance, clearly differentiated ALM and, to a lesser extent, the Cantabrian populations from the rest of the populations due to the resolutions of the *AT45m1* and *AT9* loci (Figure S7).

**Figure 5:**
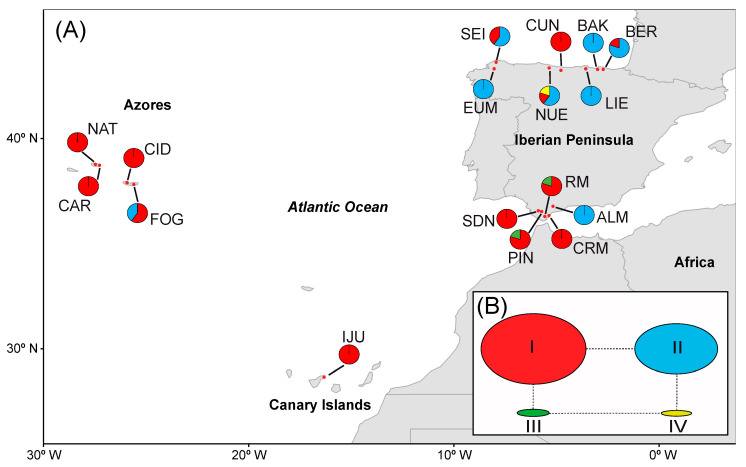
ptDNA information of the populations sampled. (**A**) Distribution of the ptDNA haplotypes (pie charts represent the frequency of each haplotype per location); (**B**) inferred ptDNA network, following the statistical parsimony method, with TCS software v1.21. Note that, in (**A**), the pie chart size is the same for all locations and does not represent the number of individuals. In (**B**), Roman numerals are the designation provided to haplotypes and circle sizes are proportional to haplotype frequencies. The color assigned to each haplotype is the same in (**A**,**B**). See Table 1 for full name of locations.

The representation on a map of the ptDNA haplotype distributions suggests a geographical structuring of them (Figure 5A). Haplotypes H-I and H-II are the most frequent and widespread, showing a generally different distribution. Haplotype H-I was dominant in Andalusia, the Canary Islands, and the Azores (called the southern supra-regional group), but it also appeared in the northern Iberian Peninsula (the Cantabrian Cornice supra-regional group) where H-II proved dominant. The latter also appeared as the only one found in ALM (Andalusia), together with H-I in FOG (the Azores). The other two minority haplotypes were exclusive to Andalusia (H-III, in PIN and RM) and to the Cantabrian Cornice (H-IV, in NUE) (Figure 5A). The ptDNA network (Figure 5B) suggested haplotype clustering in two groups (H-I and H-III; H-II and H-IV).

The AMOVA analysis of ptDNA sequences showed that, when four geographical regions were considered (Andalusia, the Azores, the Canary Islands and the Cantabrian Cornice), almost 39% (*p =* 0.0123) of variation was between regions (Table 4). With a supra-regional grouping, (southern and the Cantabrian Cornice), a differentiation between them became clear (50.53%, *p =* 0.0017; Table 4).

### 3.3. Gene Flow

The results of BAYESASS indicated no current exchange of genes with the relative exception from CAR to NAT (migration rate [*m*] = 0.1357) and from CAR to FOG (*m* = 0.1335; Table S6). The *m* estimates that did not exceed 0.110 (the upper value of the confidence interval when the data offer no information) signify no current gene flow between the populations.

The Mantel test, with and without clones, indicated a lack of isolation by distance across the populations (*r* = 0.076, *p* = 0.209 with clones; *r* = 0.055, *p* = 0.304 without clones).

### 3.4. Species Distribution Modeling

**Figure 6:**
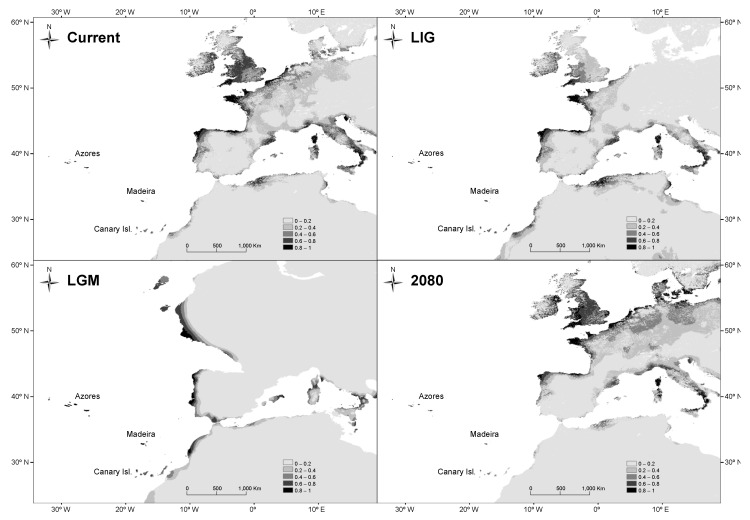
Potential distribution of *Culcita macrocarpa* drawn with MAXENT v3.4.1. (**Top left**), at the present time (Current); (**top right**), at the Last Interglacial (LIG, ca. 120,000 years BP); (**bottom left**), at the Last Glacial Maximum (LGM, ca. 21,000 years BP) using the Community Climate System Model (CCSM); (**bottom right**), prediction for the year 2080 under RCP 8.5 conditions.

For all the models, the AUC values were high (minimum value of AUC = 0.990). The MAXENT current and LIG predictions showed regions of suitable habitats that coincided largely with the species’ current distribution, with additional areas of its distribution range in the European Atlantic coasts further north and the Mediterranean Sea, where the species is currently absent (Figure 6 and Figure S8). According to LGM outputs, refugia were located in Macaronesia, the coast of Portugal, Galicia (where *C. macrocarpa* is currently present), and the European Atlantic coast (at latitudes of the present north of France and south of Great Britain, where the species is currently absent). Palaeodistribution modeling suggested no suitable habitats for *C. macrocarpa* on the northern coast of Spain (except in Galicia) where the species is currently found. The MAXENT future projections (year 2080) using the RCP8.5 scenario suggested a partial reduction in suitable habitats on the coasts of Portugal, northern Iberian Peninsula, and Macaronesian islands together with an increase in suitable habitats northward of the European Atlantic coast.

## 4. Discussion

*Culcita macrocarpa* has been regarded as one of the ferns of the herbaceous layer of European lauroid forests during the Tertiary and one that, after the geological–climatic events of the Miocene and Pliocene, survived in Macaronesian and Iberian shelters [5]. Currently, due to the reduced and fragmented nature of its distribution area, *C. macrocarpa* is considered a species under threat and protected under various European, Portuguese, and Spanish protection categories [29,30,31,32,33]. In this study, we elucidate the phylogeographic history of *C. macrocarpa* and assessed the impact of vegetative reproduction on population dynamics and genetic variability. In addition, we evaluate how the definition of “individual” chosen (ramet-based, considering all sampling units, including clonal replicates, vs. genet-based, considering only genetically distinct individuals excluding clonal replicates) affects estimates of intra-population genetic diversity indices and genetic structuring.

### 4.1. Clonality Effect on Genetic Diversity

The eight newly identified microsatellite loci had strong power to discriminate between the multilocus genotypes of *C. macrocarpa*, demonstrating their usefulness as markers for studying clonality and genetic diversity in this species. The values of the clonal descriptors discovered in the *C. macrocarpa* populations analyzed (Table 2) and AMOVA analyses (with the highest proportion of diversity found within samples, only when one MLL per individual per population was considered; Table 4) confirm the substantial overall clonality of this species. Although clonality was detected in all populations, it did not affect them equally. In general, a higher prevalence of clonality was found in Andalusian and Cantabrian populations (except three). Consequently, the impact of clonality on genetic diversity levels varied between populations. The higher the clonal prevalence, the lower the *H*_E_ and *Ar* (Figure S5). According to previous research [23], the decrease in these parameters in clonal populations may result from the absence of meiosis and recombination or interclonal competition (leading to the elimination of less-adapted clones) decreasing the amount and frequency of alleles. However, in populations tending toward strict clonality, clones will accumulate heterozygosity over time through mutation events at each locus, leading to high heterozygote excess, with each locus, in finite populations, becoming fixed for a heterozygous state [24]. Among the *C. macrocarpa* populations that were strictly clonal or that had very high clonal prevalence, many (i.e., CRM, IJU, LIE, RM, and SDN) had *H*_O_ values (although low) much higher than *H*_E_, resulting in high heterozygote excess (very negative *F*_IS_ values). On the contrary, populations such as BAK, EUM, and SEI were almost entirely or entirely homozygous, deviating from the expected pattern for strictly clonal populations. Furthermore, only one locus showed fixed heterozygosity in almost all populations, the majority being homozygous for most populations. The observed pattern in more clonal populations of *C. macrocarpa* appears to be best explained by the species’ breeding system, where intra-gametophytic selfing appears to be favored [35]. Intra-gametophytic selfing, an extreme form of inbreeding, in diploid ferns produces homozygous sporophytes at all loci. Furthermore, clonality increases self-fertilization rates, contributing to the genetic impoverishment of populations [78], also explaining the decreasing levels of genetic diversity in populations with increasing clonality.

Our results indicate that clonality affects the estimated values of genetic diversity and structuring parameters, in agreement with previous authors [49], who highlighted the risk of misinterpreting these parameters, depending on the definition of “individual” adopted (ramet-based vs. genet-based). The above study [49] focused on *H*_E_ variation, revealing that higher clonality leads to greater differences between *H*_E_ estimates of ramets and genets in a population. According to these authors, the extent and direction of this difference will depend on the size distribution of the genotypes (% of total ramets) and whether the clonal genotype is heterozygous or homozygous at the locus. In *C. macrocarpa* populations, we observed the pattern described in the above study, with variations between *H_E_* estimates increasing with higher clonality. In all cases, ramet-based *H*_E_ was lower than genet-based *H*_E_, aligning with the expected direction for the expansion of a homozygous clone according to [49]. This is consistent with the predominant homozygosity at our loci.

Concerning allelic richness (*Ar*), the observed pattern is the opposite of *H_E_*, with smaller differences between estimates with higher clonality, and consistently higher ramet-based estimates compared to genet-based estimates. *Ar* values depend heavily on the number of genes considered for rarefaction (2 × no. of individuals in the population with the fewest individuals; *g* value in [79]). As *g* decreases, *Ar* substantially reduces. In the case of clonal species such as *C. macrocarpa*, populations with very few genets will result in a significant reduction in *g* when the genet-based approach is employed to calculate *Ar*, reducing it to two in cases of monoclonal populations, as observed in our study. This factor determined the direction of the variation between estimates (ramet-based *Ar* > genet-based *Ar*). On the other hand, the smaller magnitude in the variation between estimates with increasing clonality is influenced by the effect of the decrease in the number of genes analyzed per population (*N* in [79]) and the distribution of alleles among individuals. Generally, decreases in *N* (by elimination of ramets) lead to increases in *Ar*, as occurrences of the most-frequent alleles diminish with reduced clonal redundancy, while the number of occurrences of less-frequent alleles varies minimally. The fewer alleles a locus has and the more homozygous it is (as occurs in more clonal populations), the more proportional the reduction in occurrences of the most-frequent alleles will be to the decrease in *N* and the less the occurrences of the less-frequent alleles will vary. These changes will result in higher *Ar* values, because the lower the *N*, the more the less-frequent alleles will contribute to the allelic richness of a locus. The contribution of the most-frequent alleles hardly varies (the reduction in both is proportional). Thus, in clonal populations, where a smaller number of alleles and greater homozygosity (due to the higher selfing rate), and a smaller number of different multilocus genotypes (genets) are expected, estimating *Ar* based on genets will substantially decrease *N* compared to the ramet-based approach (less pronounced in less clonal populations). Consequently, *Ar* tends to increase, partly compensating for the decrease in its value due to the smaller number of genes used for rarefaction (*g*). As a result, the difference between ramet-based and genet-based *Ar* estimates is smaller in more clonal populations. In the extreme cases of clonality (i.e., CRM, EUM, LIE, and SDN), the observed variation was minimal or non-existent.

Regarding *H*_O_ and *F*_IS_ values, differences between estimates based on the definition of “individual” were noted primarily in the most clonal populations. In these populations, the removal of clonal redundancy resulted in a higher proportion of heterozygous individuals (except in the IJU and RM populations with high *H*_O_) and correction of the *F*_IS_ value toward that of a sexual population.

Our results indicate a strong inter-population differentiation in *C. macrocarpa*. Theoretical predictions suggest that, in clonal organisms with a sexual reproduction rate, clonal reproduction tends to augment differentiation between populations compared to the parental population. This is due to the tendency for intra-population *H*_E_ to decrease without a corresponding increase in total *H*_E_, leading to higher *F*_ST_ values. However, in cases of strict clonality, populations tend to show less differentiation than sexual organisms because clonality prevents allele fixation [80]. In the case of *C. macrocarpa*, even the most strictly clonal populations exhibited very high levels of differentiation and strongly homozygous loci, contrary to expectations for strictly clonal populations. Thus, in *C. macrocarpa*, the high levels of population differentiation appear to result from the combined effect of selfing and clonality. As mentioned earlier, selfing increases homozygosity, leading to allele fixation and population differentiation, while clonality enhances the effect of selfing by boosting the rate of self-fertilization. Although the results obtained for genetic structure with ramet-based and genet-based definitions were generally congruent, the genet-based approach was less robust for detecting genetic structure in *C. macrocarpa*, showing lower levels of inter-population differentiation by eliminating clonal redundancy (Table 4 and Table S5; Figure S6).

### 4.2. Phylogeography and Population Dynamics

Due to the ability of ferns to disperse over long distances, their populations have been characterized as having low genetic differentiation, with most of the variation occurring at the intra-population level [10]. Contrary to this, our results show a global phylogeography of *C. macrocarpa* characterized by the differentiation of populations in two main groups, which coincide with the geographical distribution of the species in the northern Iberian Peninsula as well as in the Macaronesian archipelagos and the southern Iberian Peninsula. In addition to this overarching model, we have also detected a strong inter-population differentiation, even within each of the two main groups, with the absence of recent gene flow. The assessment of the contribution of individual alleles to population structuring, facilitated by DAPC analysis (Figure S7), enabled us to elucidate those that played a major role in distinguishing the two population groups and, furthermore, to provide evidence for the existence of dispersal processes, even between these groups, as indicated by the distribution pattern of these alleles across populations. A similar phylogeographic model has been described for *Vandenboschia speciosa* (Willd.) G. Kunkel (Hymenophyllaceae), a Tertiary species with a distribution similar to that of *C. macrocarpa* (although more widespread northward along the European Atlantic coast and toward central Europe), whose populations are structured in two evolutionary units, one from the north (from the Cantabrian Cornice to the north and central Europe) and another from the south (Macaronesia, Andalusia, and Italy) [19].

Integrating the results of genetic diversity, prevalence of clonal reproduction, and population differentiation paints a more complex picture than two distinct refuge regions for *C. macrocarpa*. It suggests that, within each previously proposed supra-regional group (the southern group and the Cantabrian Cornice), some populations or regions might have functioned as refugia, while others appear to be the result of post-glacial dispersal events.

A key expectation for glacial refugia is that populations persisting there for extended periods will harbor higher genetic diversity compared to recolonizing populations. These latter populations are usually composed of subsets of the genetic diversity present in the source refugial population and typically undergo founder effects and bottlenecks, culminating in reduced genetic diversity. Additionally, prolonged isolation between populations in separate refugia should lead to genetic differentiation due to genetic drift [81].

In the present study, as expected for refugia, populations in the Azores, and CUN and NUE (the Cantabrian Cornice) showed high relative genetic diversity values (Table 3; Figure S5), low clonality (Figure 2 and Figure S4A,B; Table 2), and low inter-population differentiation (Figure 3, Figure 4 and Figure S6; Tables S5 and S6). This supports the characterization of the Azores (the southern supra-regional group) and CUN/NUE (the Cantabrian Cornice supra-regional group) as refuges, at least during the Last Glacial Maximum. By contrast, the results for Andalusian populations and other Cantabrian Cornice populations follow the expectations for post-glacial dispersal events. These populations exhibited low genetic diversity (Table 3) and high clonality with a few dominant clonal lineages (Figure 2 and Figure S4A,B; Table 2). They also showed clear population differentiation (Figure 3, Figure 4 and Figure S6; Tables S5 and S6), even among geographically close populations (e.g., CRM and RM at a 2 km distance) or between the two intra-population nuclei of PIN. This model of low genetic diversity and high population differentiation has been demonstrated in populations resulting from dispersal events in several species of rock-dwelling ferns (e.g., [21,82,83]).

A prevailing assumption is that island populations exhibit lower genetic diversity compared to their mainland counterparts. However, our data from the two archipelagos studied do not support this notion. Instead, our analyses revealed that island populations show levels of genetic and genotype diversity similar to or higher than those of mainland populations (Table 2 and Table 3). Our findings add to the growing body of evidence demonstrating that island populations are not inherently less diverse (reviewed by [84]), but rather that other factors need to be considered. For instance, as in the case of *C. macrocarpa*, the role of islands as climatic refugia (where populations persist over long periods) and the ability of some plant species to disperse their propagules over long distances, as expected for spore-producing plants, (facilitating multiple colonization waves and thus secondary contact and hybridization of lineages) favor large, effective population sizes in island populations, which have been shown to be more genetically diverse than their mainland counterparts, and which can function as migratory stepping stones and can even recolonize the mainland (e.g., [19,85,86]).

The present results for species distribution modeling (SDM) suggest high suitability for *C. macrocarpa* in the Azores during the Last Interglacial (LIG) and the Last Glacial Maximum (LGM), supporting the idea of the archipelago serving as a glacial refuge for the species (Figure 6 and Figure S8). However, SDM results do not support the proposal (null suitability) that most of the Cantabrian Cornice (including CUN and NUE) acts as a glacial refuge, but rather the data suggest high suitability in the most north-westerly end of the Iberian Peninsula (Galician coast) and the Portuguese coast (Figure 6 and Figure S8). These results suggest a recolonization of the Cantabrian Cornice from those places during the Holocene. From a genetic standpoint, if this were true, we should expect the Galician populations to have the highest genetic diversity in the Cantabrian Cornice, with diversity decreasing eastward following the recolonization direction (isolation by distance model). However, our results show extremely low genetic diversity in Galician populations and, in addition, no evidence was found for isolation by distance. Our results for the Galician populations align with those of a previous study using isozymes, which reported a single multilocus genotype across all individuals form six populations [38]. To reconcile the SDM and genetic diversity results, where CUN and NUE appear to be the most diverse populations, we speculate that in the Cantabrian Cornice, populations might have persisted in small, climatically favorable pockets isolated from the prevailing climatic conditions. These populations could have then served as sources for post-glacial recolonization of the Cantabrian Cornice in various directions. A similar pattern of diversity distribution and habitat suitability in the Cantabrian Cornice was observed for the relict fern *Vandenboschia speciosa* [19].

The characteristics of *C. macrocarpa* populations with dispersal signatures are consistent with strong bottlenecks due to recent founder events by one or few genotypes, followed by expansion through vegetative reproduction. This phenomenon is known in clonal herbaceous plants (e.g., [87,88]) and particularly in ferns, where most homospore ferns are believed to establish populations from a single spore via intra-gametophytic selfing [9,82,89]. For example, this colonization strategy (single-spore colonization and subsequent population establishment) was proposed for the post-glacial colonization of Europe by *Asplenium trichomanes* subsp. *quadrivalens* D. E. Mey [21].

This colonization strategy results in a completely homozygous sporophyte derived from a single haploid gametophyte. Intra-gametophytic selfing, due to the resulting homozygosity, is predicted to have negative evolutionary consequences [90]. It reduces genetic variation within lineages, limiting their ability to adapt to changing environments. Additionally, increased homozygosity can expose partially recessive deleterious mutations, leading to decreased fertility and lower survival [90]. Therefore, to fully exploit the colonization advantage offered by intra-gametophytic selfing, species must mitigate these negative effects. Polyploidy, with its additional genome(s), has been proposed as a mechanism that may buffer against the negative genetic consequences of intra-gametophytic selfing. The additional genomes potentially prevent or delay the exposure of deleterious mutations to selection, allowing populations to persist even with selfing. Alternatively, it has been shown that intraspecific variation in mating systems might be widespread, with genotypes in isolated populations exhibiting the highest selfing capacity regardless of ploidy level [91]. The latter suggests selection for genotypes more tolerant to the detrimental effects of intra-gametophytic selfing. These tolerant genotypes would likely be the ones involved in colonization processes. Following colonization, subsequent sexual reproduction with additional immigrant genotypes is necessary to achieve increased genetic diversity and viability, especially for outcrossing diploids [90]. In this context, vegetative propagation and/or sporophytic selfing [91] could allow population expansion while prolonging the time window for the arrival of new immigrants. This strategy could explain the high diversity observed in long-lived populations, such as those in the Azores.

The highly homozygous populations EUM and BAK, and populations with near-complete homozygosity after excluding locus *CM-AT19* (with fixed heterozygosity), could be examples of colonization from a single spore followed by establishment through intra-gametophytic selfing and vegetative reproduction. However, these populations likely have not had sufficient time to receive enough immigrants to increase their genetic diversity. Our results are consistent with culture experiments demonstrating that *C. macrocarpa* gametophytes are initially male but later become hermaphroditic [34,35]. This, coupled with the lack of an antheridiogen system in *C. macrocapra*, appears to favor intra-gametophytic selfing [36,37]. This supports the notion that selection for selfing genotypes may occur during long-distance colonization events. The high homozygosity and linkage disequilibrium (based on the association index (Table 2)) detected in founded populations of *C. macrocarpa* compared to source populations further suggest differential selfing capacity, corroborating the idea raised by previous researchers [91].

The analysis of genetic diversity structure and the distribution pattern of alleles with the greatest contribution to this structure, identified through DAPC analysis, provide evidence for the occurrence of long-distance dispersal events between the two supra-regional groups identified in *C. macrocarpa*. The STRUCTURE analysis, considering all sampling units (Figure 3), revealed a third cluster linking some Cantabrian Cornice populations (BER, CUN, and NUE) with two Andalusian populations (RM and PIN) and the Canary Island population (IJU). This relationship was also reflected by the *F*_ST_ values and DAPC results (Figure 4 and Figure S7; Table S5). Notably, in the DAPC analysis, Andalusian and Canary Island populations shared alleles with high-resolution values for individual assignment to Cantabrian populations (i.e., *CM35*_106, *AT9*_267, *CM1A*_209, and *AT30*_194; Figure S7). In addition, although the two main plastid DNA (ptDNA) haplotypes (H-I and H-II) exhibit a differentiated geographical distribution, with H-I characterizing the Macaronesian group and H-II characterizing the Cantabrian group, occasional exceptions arose. The presence of H-I in the Cantabrian Cornice and H-II in Andalusia and the Azores implies discrete long-distance dispersal events.

The substructure detected within Andalusia and the contrasting nuclear and plastid affinities of its populations compared to other regions suggest multiple colonization events of this region, potentially from the Azores and the Cantabrian Cornice (or from currently extinct populations geographically closer to Andalusia). While the RM population and one intra-population nucleus of PIN share microsatellite profiles with CUN and NUE populations, they possess the typical ptDNA haplotype of the Macaronesian group. However, the presence of this ptDNA haplotype also in CUN and NUE suggests a long-distance dispersal event toward Andalusia, followed by local dispersal from RM to PIN or vice versa. Notably, PIN and RM share the only private haplotype found in Andalusia, further supporting the contention of local dispersal.

The presence of H-II in the ALM population suggests another long-distance dispersal event, potentially originating from the Cantabrian Cornice or the Azores (Figure 5A). The latter seems more likely, given the nuclear relationship of ALM with the Azores and other Andalusian populations in the STRUCTURE analysis (particularly at the uppermost hierarchical levels, *K* = 2 and *K* = 3; Figure 3) and the DAPC analysis based on Discriminant Function 1 (Figure 4). Similar results were observed in *Diplazium caudatum* (Cav.) Jermy [86], where its presence in Andalusia (its only mainland locality) was attributed to recolonization after a long-distance dispersal event from Macaronesia (presumably the Canary Islands) with subsequent establishment by a single spore or few spores through intra-gametophytic selfing and vegetative propagation, followed by local dispersals.

In the Canary Islands, only one population of *C. macrocarpa* is known. This population, with intermediate levels of genetic diversity and clonality (Table 2 and Table 3), could represent a refuge population. Alternatively, it might be a recently established population, with relatively high heterozygosity resulting from the accumulation of somatic mutations and/or additional immigrant genotypes. The limited expansion within the archipelago compared to other relict ferns sharing similar habitats (e.g., *Diplazium caudatum*, *Pteris incompleta* Cav., and *Vandenboschia speciosa*) suggests a recent arrival, although our data cannot definitively confirm this.

A limitation of this study is the lack of samples from Madeira, which would provide a more complete understanding of the connections between regions within the Macaronesian group. In a previous study on *Vandenboschia speciosa*, Madeira was found to be closely related to the southern Iberian Peninsula, while the Azores were associated with the northern evolutionary unit [19]. For *C. macrocarpa*, the Azores populations clearly relate to the south of the Iberian Peninsula and the Canary Islands. The absence of samples from Madeira hinders our ability to evaluate its potential role in facilitating the connection between the Azores, the Canary Islands, and the southern Iberian Peninsula.

### 4.3. Conservation and Future Perspectives

The identification of two supra-regional groups (the Cantabrian cornice/South) could help design a management plan to improve conservation measures. This fern is especially threatened in Andalusia, where only haplotypes and alleles are present, making it critical to protect this area. In relation to predictions, SDM results in 2080 show a heavy loss of habitat suitability for *C. macrocarpa* in Andalusia and in the Canary Islands, and also for the north-western Iberian Peninsula, but a gain in habitat suitability toward northern Europe (Figure 6 and Figure S8). All this warns of a potential threat to the populations of the southern group and implies a northward migration of the species, necessitating the protection of the southern populations in an effort to prevent the loss of genetic diversity.

The results regarding the variation in the estimates of genetic diversity and genetic structuring descriptors, depending on the adopted definition of “individual”, emphasize the need for caution in interpreting the estimates when formulating management and conservation measures for endangered species with clonal propagation. The choice between genet-based and ramet-based definitions can result in an under- or over-estimation of these parameters. Following the recommendation of other authors [49], in cases where the vegetative propagation of the species is known, parameters should be adopted using both approaches.

## Figures and Tables

**Table 1:** Sampling details of *Culcita macrocarpa* populations used in the present study.

				Sample Size	
Code	Location	Voucher	Geographical Coordinates	Microsatellites	ptDNA
Andalusia					
ALM	Cádiz: Almoraima	GDA 65361	N 36.304°/W 5.520°	6	3
CRM	Cádiz: Cabecera del río de la Miel	GDA 65363	N 36.105°/W 5.528°	30	5
PIN	Cádiz: Laja del Pinarejo	GDA 65360	N 36.188°/W 5.589°	30	5
RM	Cádiz: Río de la Miel	GDA 65359	N 36.112°/W 5.507°	29	5
SDN	Cádiz: Sierra del Niño	GDA 65362	N 36.186°/W 5.610°	30	5
Azores					
CAR	Terceira: Algar do Carvão	GDA 63533	N 38.727°/W 27.215°	30	5
CID	São Miguel: Sete Cidades	GDA 63534	N 37.835°/W 25.788°	29	5
FOG	São Miguel: Lagoa do Fogo	GDA 63532		30	5
NAT	Terceira: Gruta do Natal	GDA 63531	N 38.738°/W 27.264°	30	5
Canary Isl.					
IJU	Tenerife: Ijuana	GDA 63536	N 28.560°/W 16.172°	30	4
Cantabrian Cornice					
BER	Bizkaia: Bermeo	GDA 63539	N 43.392°/W 2.734°	12	5
BAK	Bizkaia: Bakio	GDA 65364	N 43.425°/W 2.845°	18	5
CUN	Asturias: San Esteban de Cuñaba	GDA 63537	N 43.277°/W 4.676°	30	5
EUM	A Coruña: Eume	GDA 63535	N 43.404°/W 8.087°	30	5
LIE	Cantabria: Liendo	GDA 65365	N 43.375°/W 3.383°	30	5
NUE	Asturias: Nueva de Llanes	GDA 63538	N 43.421°/W 4.954°	30	5
SEI	A Coruña: Seixo	GDA 63530	N 43.706°/W 7.946°	19	5

GDA, University of Granada herbarium.

**Table 2:** Clonality descriptors in the populations of *Culcita macrocarpa* studied. Descriptors were separated into clonal richness, genotype diversity, and linkage disequilibrium. N, number of individuals sampled. MLL, number of different multilocus lineages, or clonal lineages, with exclusive multilocus lineages in brackets; eMLL, number of expected multilocus lineages at the smallest sample size ≥ 6 based on rarefaction [70]; R, clonal richness [71]; lambda, Simpson’s index [72]; E.5, evenness [73,74,75]; *r*_d_, standardized index of association [45]; *gr*_d_, *r*_d_ calculated considering only one individual per multilocus lineage per population (genet-based calculation). See Table 1 for full name of locations.

		Clonal Richness			Genotype Diversity		Linkage Disequilibrium	
Population	N	MLL (Private)	eMLL	R	Lambda	E.5	*r*_d_	*gr*_d_
Andalusia	125	16 (12)	6.56	0.121	0.579	0.464	0.402 *	0.186 *
ALM	6	4 (4)	4	0.6	0.8	0.812	NA	NA
CRM	30	2	1.33	0.034	0.067	0.438	NA	NA
PIN	30	8 (5)	4.12	0.241	0.623	0.528	0.728 *	0.193 *
RM	29	3 (3)	1.92	0.071	0.197	0.48	0.141 *	0.5
SDN	30	1	1	0	0	NA	NA	NA
Azores	119	35 (25)	16.01	0.288	0.904	0.5	0.041 *	−0.05
CAR	30	12 (4)	6.53	0.379	0.881	0.727	0.062 *	−0.047
CID	29	14 (12)	6.98	0.464	0.901	0.719	0.140 *	0.003
FOG	30	12 (5)	6.68	0.379	0.885	0.725	0.029	−0.063
NAT	30	11 (4)	6.03	0.345	0.855	0.713	0.005	−0.067
Canary Isl.	30	7 (5)	7	0.207	0.811	0.836	−0.018	−0.248
IJU	30	7 (5)	4.86	0.207	0.811	0.836	−0.018	−0.248
Cantabrian Cornice	169	53 (44)	14.93	0.31	0.853	0.347	0.185 *	0.085 *
BER	12	6 (6)	5.33	0.454	0.803	0.762	0.3 *	−0.042
BAK	18	3 (2)	2.55	0.118	0.503	0.757	0.536 *	0
CUN	30	23 (16)	9.2	0.759	0.982	0.913	0.04 *	0.011
EUM	30	1	1	0	0	NA	NA	NA
LIE	30	1 (1)	1	0	0	NA	NA	NA
NUE	30	18 (16)	8.01	0.586	0.942	0.76	0.07 *	0.024
SEI	19	4 (3)	2.84	0.166	0.38	0.52	0.725 *	0.12
Total	443 (130)	104 (91)	7.69	0.292	0.926	0.384	0.288	

* *p* < 0.05; NA, not applicable.

**Table 3:** Genetic diversity indices for microsatellites and ptDNA sequences in the populations of *Culcita macrocarpa* studied. Indices were calculated including all individuals sampled per population (ramet-based calculation) and including only one individual per multilocus lineage per population (gIndex; genet-based calculation). N, number of individuals sampled; *A*, number of alleles with unique alleles in brackets; *Ar*, allelic richness at the smallest sample size (12 and 2 for populations with clones and without clones, respectively; 60 and 12 for geographical regions with clones and without clones, respectively) based on rarefaction; *H*_O_, observed heterozygosity; *H*_E_, expected heterozygosity [76]; *F*_IS_, inbreeding coefficient [77]; *ha*, number of haplotypes with unique haplotypes in brackets; *Hd*, haplotype diversity; *π*, nucleotide diversity (×10^2^). See Table 1 for full name of locations.

		Microsatellites									ptDNA		
Population	N	*A* (Private)	*Ar*	*gAr*	*H*_O_	*gH*_O_	*H*_E_	*gH*_E_	*F*_IS_	*gF*_IS_	*ha*	*Hd*	*π*
Andalusia	125	18 (3)	2.228	2.187	0.164	0.215	0.331	0.426	0.505 *	0.494 *	3 (1)	0.38	0.17
ALM	6	11 (1)	1.375	1.165	0.146	0.156	0.146	0.167	0.000	0.063	1	0.00	0.00
CRM	30	10	1.15	1.146	0.129	0.188	0.067	0.125	−0.938 *	−0.500	1	0.00	0.00
PIN	30	12	1.498	1.255	0.163	0.250	0.232	0.267	0.301 *	0.063	2	0.40	0.17
RM	29	11 (1)	1.338	1.217	0.246	0.250	0.145	0.208	−0.692 *	−0.200	2	0.40	0.17
SDN	30	9	1.125	1.125	0.125	NA	0.063	NA	−1.000 *	NA	1	0.00	0.00
Azores	119	21 (6)	2.414	2.159	0.169	0.181	0.267	0.330	0.366 *	0.452 *	2	0.19	0.08
CAR	30	15 (1)	1.708	1.307	0.175	0.177	0.235	0.308	0.257 *	0.425 *	1	0.00	0.00
CID	29	17 (2)	1.932	1.365	0.198	0.205	0.327	0.371	0.394 *	0.447 *	1	0.00	0.00
FOG	30	16 (1)	1.727	1.280	0.163	0.188	0.232	0.284	0.299 *	0.340 *	2	0.60	0.25
NAT	30	15	1.668	1.273	0.142	0.148	0.209	0.280	0.323 *	0.472 *	1	0.00	0.00
Canary Isl.	30	13	1.625	1.625	0.267	0.268	0.193	0.211	−0.385 *	−0.268	1	0.00	0.00
IJU	30	13	1.498	1.216	0.267	0.268	0.193	0.211	−0.385 *	−0.268	1	0.00	0.00
Cantabrian Cornice	169	26 (8)	2.745	2.461	0.133	0.248	0.369	0.473	0.639 *	0.476 *	3 (1)	0.42	0.18
BER	12	16 (1)	1.815	1.333	0.125	0.208	0.288	0.346	0.566 *	0.398 *	2	0.40	0.17
BAK	18	11	1.320	1.2	0.000	0.000	0.132	0.250	1.000 *	1.000 *	1	0.00	0.00
CUN	30	16 (1)	1.879	1.373	0.425	0.408	0.364	0.372	−0.169 *	−0.096	1	0.00	0.00
EUM	30	8	1	1	0.000	NA	0.000	NA	NA	NA	1	0.00	0.00
LIE	30	9	1.125	1.125	0.125	NA	0.063	NA	−1.000 *	NA	1	0.00	0.00
NUE	30	19 (1)	2.084	1.341	0.117	0.132	0.347	0.356	0.663 *	0.630 *	3 (1)	0.70	0.34
SEI	19	16 (1)	1.605	1.339	0.053	0.188	0.149	0.365	0.646 *	0.486 *	2	0.60	0.25
Total	443	37	1.520	1.239	0.153	0.177	0.528	0.545	0.186 *	0.357 *	4	0.52	0.0023

* *p* < 0.05; NA, not applicable.

**Table 4:** Hierarchical analysis of molecular variance (AMOVA). d.f., degree of freedom; MLLs, analyses including only one individual per multilocus lineage per population.

Source of Variation	d.f.	Sum of Squares	Percentage of Variation	Phi	*p*-Value
***Microsatellites***					
**All sampling units** (ramet-based analyses)					
*Standard*					
Within samples	443	547	29,23	0.708	<0.001
Between samples within populations	426	742.883	6.03	0.171	<0.001
Between populations	16	2296.697	64.74	0.647	<0.001
Total	885	3586.58	100		
*Hierarchical* (4 geographical regions)					
Within samples	443	547	26.41	0.736	<0.001
Between samples within populations	426	742.883	5.44	0.171	<0.001
Between population within regions	13	949.498	30.18	0.486	<0.001
Between regions	3	1347.199	37.97	0.379	<0.001
Total	885	3586.58	100		
**MLLs** (genet-based analyses)					
*Standard*					
Within samples	130	224	42.6	0.574	<0.001
Between samples within populations	113	371.188	19.31	0.312	<0.001
Between populations	16	413.658	38.09	0.381	<0.001
Total	259	1008.846	100		
*Hierarchical* (4 geographical regions)					
Within samples	130	224	40.44	0.596	<0.001
Between samples within populations	113	371.188	18.33	0.312	<0.001
Between population within regions	13	217.449	23.02	0.281	<0.001
Between regions	3	196.208	18.21	0.182	<0.001
Total	259	1008.846	100		
***ptDNA***					
*Standard*					
Between populations	17	16.529	65.96		<0.001
Within populations	66	6.4	34.04		<0.001
Total	83	22.929	100		
*Hierarchical* (4 geographical regions)					
Between regions	3	8.069	38.37		0.0123
Between populations within regions	14	7.314	30.44		<0.001
Within populations	66	6.200	31.19		<0.001
Total	83	21.583	100		
*Hierarchical* (2 supra-regional groups)					
Between groups	1	7.873	50.53		0.0017
Between populations within groups	16	7.511	22.87		<0.001
Within populations	66	6.200	26.60		<0.001
Total	83	21.583	100		

## Data Availability

All sequence data obtained in this study are available from the GenBank database (microsatellite accession numbers: OR965870–OR965877; ptDNA accession numbers: OR965866–OR965869).

## Supplementary Materials

The following supporting information can be downloaded at: https://www.mdpi.com/article/10.3390/plants13121587/s1, Table S1: Characteristics of eight microsatellite loci developed in *Culcita macrocarpa*; Table S2: Primer pairs used for PCR amplification of the 13 ptDNA regions tested in *Culcita macrocarpa*; Table S3: Percentage contribution and permutation importance (MaxEnt) of selected model for the species distribution modeling (SDM); Table S4: Values for *F*_IS_ per population and per locus with and without locus *CM-AT19*; Table S5: Pairwise population *F*_ST_ for microsatellites; Table S6: Mean recent migration rates (*m*) among the studied populations, estimated from eight microsatellite loci using the BAYESASS program; Figure S1: Location of presence records used for species distribution modeling (SDM); Figure S2: Genotypic accumulation curve showing the resolutive power of the eight microsatellites used in this study; Figure S3: Histogram of frequency distribution of pairwise genetic distances; Figure S4: Distribution of the 104 MLLs among the 130 individuals (genets) of *Culcita macrocarpa*; Figure S5: Scatterplots of genetic diversity estimates, obtained with eight microsatellite loci, against clonal richness (R) of *Culcita macrocarpa* populations; Figure S6: Bar plots showing the STRUCTURE results, using microsatellite data and assuming the no-admixture model; Figure S7: Additional results of the discriminant analysis of principal components (DAPC); Figure S8: Detailed potential distribution of *Culcita macrocarpa* in the Iberian Peninsula and Macaronesian Islands drawn with MAXENT. Refs. [92,93] are cited in the Supplementary Materials.
