# Supplementary material for: Genetic Diversity and Phylogeography of the Relict Tree Fern Culcita macrocarpa: Influence of Clonality and Breeding System on Genetic Variation
Source: Plants (Basel). 2024 Jun 7;13(12):1587. doi: 10.3390/plants13121587 (PMC11207926; doi:10.3390/plants13121587)
Supplement: Supplementary file 1 [file plants-13-01587-s001.zip › plants-2985952-supplementary.pdf]

Supplementary Material

Genetic Diversity and Phylogeography of the Relict Tree Fern *Culcita macrocarpa*: Influence of Clonality and Breeding System on Genetic Variation

Víctor N. Suárez-Santiago, Jim Provan, Ana Teresa Romero-García and Samira Ben-Menni Schuler

The following Supplementary data are available for this article:

Table S1. Characteristics of eight microsatellite loci developed in *Culcita macrocarpa*.

Table S2. Primer pairs used for PCR amplification of the 13 ptDNA regions tested in *Culcita macrocarpa*.

Table S3. Percentage contribution and permutation importance (MaxEnt) of selected model for the species distribution modelling (SDM).

Table S4. Values for F_{IS} per population and per locus with and without locus *CM-AT19*.

Table S5. Pairwise population F_{ST} for microsatellites.

Table S6. Mean recent migration rates (*m*) among the studied populations, estimated from eight microsatellite loci using the BAYESASS program.

Figure S1. Location of presence records used for species distribution modelling (SDM).

Figure S2. Genotypic accumulation curve showing the resolutive power of the eight microsatellite used in this study.

Figure S3. Histogram of frequency distribution of pairwise genetic distances

Figure S4. Distribution of the 104 MLLs among the 130 individuals (genets) of *Culcita macrocarpa*.

Figure S5. Scatterplots of genetic diversity estimates, obtained with eight microsatellite loci, against clonal richness (R) of *Culcita macrocarpa* populations.

Figure S6. Bar plots showing the STRUCTURE results, using microsatellite data and assuming the non admixture model.

Figure S7. Additional results of the discriminant analysis of principal components (DAPC).

Figure S8. Detailed potential distribution of *Culcita macrocarpa* in the Iberian Peninsula and Macaronesian Islands drawn with MAXENT.

Table S1. Characteristics of eight microsatellite loci developed in *Culcita macrocarpa*.

Locus	Primer sequences (5′–3′)[a]	Repeat motif	Allele size range (bp)	Bet (M)	$MgCl_2$ (mM)	T_a (°C)[b]	GenBank accession no.
CM-1'A	F:TTAAGGTCAAACTACCACCA<br>R:GTTTACCCAATTGTTTAACTGTTC	(GT)8	207-209	1	1.25	D60-50	OR965870
CM-35	F:GGATATCATTTCAGATGCAC<br>R:GTTTAGCACATGTAGCTAATC	(AG)17	106-110	1	1.25	D60-50	OR965872
CM-21b	F:GTATGCCGCACCTAATAAAG<br>R:GTTTACTTCGCGCTATGAAATACT	(GT)7	204-206	1	1.25	D60-52	OR965871
CM-AT30	F:CCGAGAAGGCCATGTGTAAG<br>R:GTTTCCATGCAAAAAGAAGGGACA	(GA)15	178-196	0.5	0.25	58	OR965875
CM-AT45m1	F:CATGGCTATGGTGTTTGGAGA<br>R:GTTTTGAGTCGTGCAATACTTGTCTC	(AG)22	126-142	0.5	0	60	OR965876
CM-AT9	F:GGGGATTACTGGCTATACTG<br>R:GTTTCTCGGAGATTTATATGCTTG	(TC)26	261-267	0	0	60	OR965877
CM-AT2	F:AAGAGTCGTCCAACACTAATTCT<br>R: AGAAAGAAAGAGGAGTCACGA	(CT)26	140-174	0.5	0.25	55	OR965874
CM-AT19	F:CCAACTGTACGAGGAGCACA<br>R:GTTTAGGAAGTTCAGGGTTATGG	(TTTC)2(TC)7CG(TC)22	268-288	0	0	62	OR965873

Note: Bet = optimal betaine concentration; MgCl2 = optimal magnesium chloride concentration; Ta = annealing temperature. Underlined nucleotides in reverse primers indicates a tail added to the original primer to improve PCR reactions.

[a] Forward primer sequence and allele size range do not include the M13-tail sequence (5′-CACGACGTTGTAAAACGAC-3′).

[b] Cycling parameters: 94°C 3min, followed by 35 cycles of 94°C 1 min, Ta 1 min, and 72°C 45 s; and a final extension at 72°C 20 min. D60: inicial step of 5 cycles with Ta at 60 °C for 5 min before initiating the 35 cycles. PCR reactions: 10-µL containing 20 ng of genomic DNA, 0.02 µM of the M13-labeled forward primer, 0.45 µM of each reverse primer and fluorolabeled M13 primer (5′-6FAM, 5′-HEX, 5′-ATTO-550), 0–1.25 mM MgCl2, 0.5 mM dNTPs, 0–1 M Betaine, 1 µL Biotools 10× Reaction Buffer, and 0.5 units of Biotools DNA polymerase (Biotools B&M Laboratories S.A., Madrid, Spain).

Table S2. Primer pairs used for PCR amplification of the 13 ptDNA regions tested in *Culcita macrocarpa.*

[a] Primers without reference were designed from the partial chloroplast genome of *Plagiogyria glauca* (Blume) Mett. (GenBank accession number: KP136831).

Primer	Sequences 5′-3′	Region[a]
rpL16-F-Culcit	ATGCCTAGTGTGCGACCCGTT	*rpl16* intron
rpL16-R-Culcit	TCCTCTATGTTGCTTACGGAAT	*rpl16* intron
CM-rpS16-F	AAGCGACATGGTGGGAAGCAA	*rps16* intron
CM-rpS16-R	CGGGACCGAGCATCAATTGCTA	*rps16* intron
CM-ycf3F	GCTTCTACATATTATAGGGATG	*ycf3* introns
CM-ycf3R	TTGAATGGCCTGTTCCCC	*ycf3* introns
trnL(UAG)	CTGCTTCCTAAGAGCAGCGT	*rpl32-trnL*
rpL32-F	CAGTTCCAAAGAAACGTACCTC	*rpl32-trnL*
CM-rps4-trnL(CAA)-Fw	CTTCTCCTGGATTGGATGCT	*rps4-trnL* spacer
CM-rpS4-trnL(CAA)-Rv	CGCAGCGTCTACCGTTCCG	*rps4-trnL* spacer
CM-rps16-matK-Fw	CATATTGAAACGGGCAAAGG	*rps16-matK*
CM-rps16-matK-Rev	CTTTTCCGTAACGTCCCAAA	*rps16-matK*
CM-trnD-psbM-F	ACCGATTGAACTACAATCCC	*trnD*(GUC)-*psbM*
CM-trnD-psbM-R	GGCTACGAACGCAAGAATATTGACTTCCGT	*trnD*(GUC)-*psbM*
CM-trnD(GUC)-R	GGGATTGTAGTTCAATCGGT	*trnD-trnY-trnE-rpoB*
CM-rpoB	CGGTTAGATCCCTCAAATTG	*trnD-trnY-trnE-rpoB*
CM-trnH(GUG)2	TGGATCCACAATCCATTGC	*trnH-psbA*
CM-psbA2	CGTAACGCTCATAACTTCCCTCT	*trnH-psbA*
f	ACTTGAACTGGTGACACGAG	*trnL-trnL-trnF*; [1]
e	GGTTCAAGTCCCTCTATCCC	*trnL-trnL-trnF*; [1]
CM-trnL-Fw	CGGAACGGTAGACGCTGCG	*trnL-trnL-trnF*
trnS(GCU)	AGAGAGGGATTCGAACCCTCGGT	*trnS-trnG-trnG*; [2]
3′trnG(UUC)	GTAGCGGGAATCGAACCCGCATC	*trnS-trnG-trnG*; [2]
5′trnG2S	TTTACCACTAAACCATACCCGC	*trnS-trnG*; [2]
5′trnG2G	GCGGGTATGGTTTAGTGGTAAAA	*trnG-trnG*; [2]

Table S3. Percent contribution and permutation importance (MaxEnt) of selected model for the species distribution modelling (SDM).

Variable[a]	MaxEnt Percent contribution	MaxEnt Permutation importance
**Mean Diurnal Range**	48	11
**Min. Temperature of Coldest Month**	30.9	80.1
Type of Soil	10.6	1.4
Precipitation of Warmest Quarter	8.4	5
Precipitation of Coldest Quarter	1	0.1
Max. Temperature of Warmest Month	0.7	2
Precipitation Seasonality	0.3	0.4
Precipitation of Wettest Month	0.1	0.2
Precipitation of Driest Month	0	0

[a] Variables in bold were selected for the final model.

Table S4. Values for F_{IS} per population and per locus with and without locus *CM-AT19*. For population codes, see Table 1.

	With *CM-AT19*									Without *CM-AT19*							
**Population**	***CM1A***	***CM35***	***CM21b***	***CM-AT30***	***CM-AT45m1***	***CM-AT9***	***CM-AT2***	***CM-AT19***	**Multi-locus**	***CM1A***	***CM35***	***CM21b***	***CM-AT30***	***CM-AT45m1***	***CM-AT9***	***CM-AT2***	**Multi-locus**
CRM	---	---	---	---	---	---	0.000	-1.000	-0.938*	---	---	---	---	---	---	0.000	0.000
PIN	---	---	---	0.776	---	0.861	0.691	-1.000	0.301*	---	---	---	0.776	---	0.861	0.691	0.779*
ALM	---	---	---	---	---	---	0.750	-1.000	0.000	---	---	---	---	---	---	0.750	0.750*
RM	---	---	-0.867	---	---	---	0.788	-1.000	-0.692*	---	---	-0.867	---	---	---	0.788	-0.460*
SDN	---	---	---	---	---	---	---	-1.000	-1.000*	---	---	---	---	---	---	---	---
CID	---	1.000	0.154	0.729	---	1.000	0.853	-1.000	0.394*	---	1.000	0.154	0.729	---	1.000	0.853	0.723*
NAT	---	---	1.000	---	1.000	1.000	0.779	-1.000	0.323*	---	---	1.000	---	1.000	1.000	0.779	0.887*
CAR	---	---	0.437	---	0.846	1.000	0.557	-1.000	0.257*	---	---	0.437	---	0.846	1.000	0.557	0.711*
FOG	---	---	-0.036	0.728	1.000	1.000	0.721	-1.000	0.299*	---	---	-0.036	0.728	1.000	1.000	0.721	0.779*
EUM	---	---	---	---	---	---	---	---	---	---	---	---	---	---	---	---	---
SEI	1.000	---	0.854	0.786	-0.029	0.000	---	-0.059	0.646*	1.000	---	0.854	0.786	-0.029	0.000	---	0.747*
IJU	---	---	---	0.120	---	-0.383	---	-1.000	-0.385*	---	---	---	0.120	---	-0.383	---	-0.090
BER	0.656	---	---	1.000	0.560	---	0.814	-0.375	0.566*	0.656	---	---	1.000	0.560	---	0.814	0.778*
BAK	1.000	---	---	1.000	---	---	1.000	---	1.000*	1.000	---	---	1.000	---	---	1.000	1.000*
LIE	---	---	---	---	---	---	---	-1.000	-1.000*	---	---	---	---	---	---	---	---
NUE	1.000	0.439	0.000	0.630	0.870	0.867	1.000	-0.137	0.663*	1.000	0.439	0.000	0.630	0.870	0.867	1.000	0.737*
CUN	0.000	-0.674	0.164	0.100	0.138	0.065	---	-0.758	-0.169*	0.000	-0.674	0.164	0.100	0.138	0.065	---	-0.049
Overall	0.898	0.125	0.085	0.634	0.609	0.636	0.748	-0.894	0.186	0.898	0.125	0.085	0.634	0.609	0.636	0.748	0.578

* $P < 0.05$

Table S5. Pairwise population F_{ST} for microsatellites. F_{ST} values with all sampled individuals are represented below de diagonal, and F_{ST} values with a single representing multilocus lineage per population above the diagonal. Values in bold were significant at the 5% nominal level after sequential Bonferroni correction. For population codes, see Table 1.

	CRM	PIN	ALM	RM	SDN	CID	NAT	CAR	FOG	EUM	SEI	IJU	BER	BAK	LIE	NUE	CUN
CRM	--	0.211	0.611	0.700	-	0.000	-0.043	-0.031	-0.076	-	0.589	0.485	0.591	0.767	-	0.442	0.264
PIN	**0.188**	--	0.513	0.573	-	0.210	**0.176**	**0.197**	0.180	-	0.523	**0.204**	**0.515**	0.688	-	**0.446**	**0.251**
ALM	**0.800**	**0.529**	--	0.770	-	**0.439**	0.404	**0.402**	0.407	-	0.638	0.600	0.650	0.781	-	**0.533**	**0.435**
RM	**0.830**	**0.679**	**0.820**	--	-	**0.471**	**0.599**	**0.574**	**0.570**	-	0.504	**0.658**	0.595	0.686	-	**0.525**	**0.447**
SDN	0.000	**0.196**	**0.809**	**0.834**	--	-	-	-	-	-	-	-	-	-	-	-	-
CID	**0.330**	**0.187**	**0.473**	**0.608**	**0.339**	--	0.120	0.109	0.081	-	**0.490**	**0.306**	**0.497**	0.617	-	**0.380**	**0.236**
NAT	**0.193**	0.093	**0.517**	**0.709**	**0.204**	**0.137**	--	-0.030	0.024	-	0.566	**0.244**	**0.568**	0.699	-	**0.425**	**0.247**
CAR	**0.326**	**0.164**	**0.482**	**0.684**	**0.338**	**0.119**	0.025	--	0.013	-	**0.541**	**0.247**	**0.536**	0.667	-	**0.380**	**0.224**
FOG	**0.259**	**0.126**	**0.472**	**0.679**	**0.272**	**0.109**	0.027	0.022	--	-	0.547	**0.277**	**0.570**	0.692	-	**0.413**	**0.203**
EUM	**0.964**	**0.865**	**0.972**	**0.875**	**0.967**	**0.810**	**0.881**	**0.860**	**0.864**	--	-	-	-	-	-	-	-
SEI	**0.888**	**0.754**	**0.818**	**0.744**	**0.891**	**0.687**	**0.777**	**0.748**	**0.753**	0.137	--	**0.553**	0.434	0.227	-	0.237	**0.256**
IJU	**0.683**	**0.407**	**0.616**	**0.717**	**0.689**	**0.384**	**0.427**	**0.366**	**0.406**	**0.864**	**0.740**	--	**0.578**	0.737	-	**0.443**	**0.261**
BER	**0.819**	**0.638**	**0.696**	**0.738**	**0.823**	**0.567**	**0.678**	**0.646**	**0.657**	**0.756**	**0.530**	**0.650**	--	0.367	-	**0.396**	**0.474**
BAK	**0.903**	**0.777**	**0.842**	**0.776**	**0.906**	**0.711**	**0.798**	**0.771**	**0.776**	0.349	0.126	**0.773**	**0.523**	--	-	0.316	**0.496**
LIE	**0.920**	**0.809**	**0.895**	**0.820**	**0.923**	**0.753**	**0.820**	**0.791**	**0.802**	**0.833**	**0.587**	**0.830**	**0.710**	**0.616**	--	-	-
NUE	**0.668**	**0.520**	**0.552**	**0.608**	**0.673**	**0.413**	**0.514**	**0.459**	**0.487**	**0.569**	**0.389**	**0.484**	**0.434**	**0.403**	**0.528**	--	**0.188**
CUN	**0.496**	**0.307**	**0.438**	**0.530**	**0.504**	**0.222**	**0.286**	**0.225**	**0.231**	**0.688**	**0.517**	**0.317**	**0.512**	**0.565**	**0.621**	**0.214**	--

Table S6. Mean recent migration rates (m) among the studied populations, estimated from eight microsatellite loci using the BAYESASS program. Values on the diagonal (underlined) indicate the proportion of individuals in each generation that are not migrants. Values in bold are the m rates that are informative. For population codes, see Table 1.

From / To	CRM	PIN	ALM	RM	SDN	CID	NAT	CAR	FOG	EUM	SEI	IJU	BER	BAK	LIE	NUE	CUN
CRM	0.684 (0.016)	0.017 (0.017)	0.018 (0.017)	0.016 (0.016)	0.016 (0.016)	0.018 (0.016)	0.02 (0.019)	0.048 (0.027)	0.017 (0.016)	0.017 (0.015)	0.018 (0.018)	0.017 (0.017)	0.018 (0.017)	0.016 (0.015)	0.019 (0.017)	0.017 (0.016)	0.016 (0.015)
PIN	0.013 (0.014)	0.680 (0.013)	0.013 (0.012)	0.014 (0.013)	0.013 (0.013)	0.013 (0.012)	0.015 (0.014)	0.038 (0.022)	0.013 (0.013)	0.013 (0.012)	0.013 (0.012)	0.092 (0.031)	0.012 (0.012)	0.013 (0.012)	0.012 (0.011)	0.014 (0.013)	0.013 (0.013)
ALM	0.015 (0.015)	0.015 (0.014)	0.745 (0.030)	0.015 (0.014)	0.016 (0.015)	0.016 (0.016)	0.017 (0.016)	0.014 (0.014)	0.015 (0.013)	0.015 (0.015)	0.016 (0.016)	0.016 (0.017)	0.016 (0.015)	0.015 (0.013)	0.015 (0.014)	0.015 (0.015)	0.016 (0.015)
RM	0.0154 (0.015)	0.015 (0.014)	0.014 (0.014)	0.756 (0.030)	0.015 (0.015)	0.014 (0.012)	0.015 (0.013)	0.015 (0.015)	0.014 (0.015)	0.014 (0.014)	0.016 (0.015)	0.015 (0.014)	0.015 (0.015)	0.015 (0.015)	0.015 (0.014)	0.015 (0.015)	0.015 (0.013)
SDN	0.018 (0.017)	0.017 (0.016)	0.018 (0.016)	0.018 (0.017)	0.685 (0.017)	0.017 (0.016)	0.021 (0.02)	0.034 (0.024)	0.018 (0.017)	0.019 (0.017)	0.018 (0.018)	0.018 (0.018)	0.018 (0.016)	0.018 (0.016)	0.018 (0.016)	0.019 (0.018)	0.018 (0.017)
CID	0.009 (0.008)	0.009 (0.009)	0.010 (0.011)	0.010 (0.010)	0.01 (0.01)	0.749 (0.029)	0.013 (0.014)	0.094 (0.030)	0.009 (0.009)	0.01 (0.009)	0.01 (0.01)	0.01 (0.01)	0.009 (0.009)	0.011 (0.01)	0.011 (0.01)	0.01 (0.012)	0.01 (0.009)
NAT	0.011 (0.010)	0.011 (0.011)	0.012 (0.012)	0.012 (0.011)	0.012 (0.012)	0.012 (0.012)	0.685 (0.02)	**0.135** (0.033)	0.011 (0.011)	0.011 (0.01)	0.011 (0.011)	0.013 (0.013)	0.011 (0.011)	0.012 (0.012)	0.013 (0.012)	0.011 (0.011)	0.012 (0.011)
CAR	0.010 (0.010)	0.01 (0.01)	0.011 (0.010)	0.010 (0.010)	0.01 (0.009)	0.012 (0.012)	0.014 (0.015)	0.819 (0.032)	0.011 (0.011)	0.01 (0.009)	0.009 (0.009)	0.012 (0.012)	0.011 (0.01)	0.012 (0.01)	0.01 (0.011)	0.011 (0.011)	0.012 (0.011)
FOG	0.011 (0.011)	0.011 (0.01)	0.011 (0.010)	0.010 (0.010)	0.011 (0.011)	0.014 (0.012)	0.023 (0.027)	**0.133** (0.035)	0.678 (0.011)	0.011 (0.010)	0.011 (0.01)	0.012 (0.011)	0.011 (0.011)	0.011 (0.011)	0.011 (0.01)	0.011 (0.011)	0.011 (0.01)
EUM	0.019 (0.018)	0.018 (0.016)	0.018 (0.018)	0.020 (0.019)	0.019 (0.017)	0.017 (0.017)	0.016 (0.016)	0.018 (0.018)	0.019 (0.018)	0.686 (0.018)	0.018 (0.017)	0.018 (0.016)	0.019 (0.017)	0.024 (0.021)	0.018 (0.017)	0.029 (0.023)	0.018 (0.016)
SEI	0.018 (0.018)	0.016 (0.015)	0.016 (0.015)	0.016 (0.015)	0.015 (0.014)	0.016 (0.016)	0.015 (0.015)	0.016 (0.015)	0.017 (0.019)	0.015 (0.014)	0.684 (0.016)	0.015 (0.015)	0.015 (0.014)	0.031 (0.025)	0.016 (0.015)	0.025 (0.022)	0.047 (0.027)
IJU	0.012 (0.011)	0.012 (0.011)	0.012 (0.011)	0.012 (0.011)	0.012 (0.011)	0.011 (0.01)	0.012 (0.012)	0.018 (0.015)	0.012 (0.010)	0.011 (0.01)	0.011 (0.011)	0.799 (0.032)	0.012 (0.012)	0.011 (0.011)	0.011 (0.01)	0.012 (0.012)	0.015 (0.014)
VER	0.014 (0.013)	0.014 (0.014)	0.014 (0.013)	0.014 (0.014)	0.014 (0.013)	0.015 (0.015)	0.014 (0.013)	0.014 (0.014)	0.014 (0.014)	0.015 (0.015)	0.016 (0.016)	0.015 (0.014)	0.753 (0.032)	0.02 (0.019)	0.016 (0.015)	0.016 (0.015)	0.015 (0.014)
BAK	0.017 (0.016)	0.016 (0.016)	0.015 (0.014)	0.017 (0.017)	0.015 (0.014)	0.015 (0.015)	0.018 (0.017)	0.016 (0.015)	0.016 (0.015)	0.018 (0.016)	0.018 (0.017)	0.015 (0.015)	0.017 (0.016)	0.712 (0.032)	0.016 (0.016)	0.035 (0.031)	0.018 (0.016)
LIE	0.019 (0.017)	0.018 (0.017)	0.018 (0.017)	0.018 (0.017)	0.017 (0.016)	0.019 (0.018)	0.018 (0.018)	0.018 (0.017)	0.017 (0.015)	0.018 (0.017)	0.018 (0.017)	0.017 (0.015)	0.019 (0.017)	0.024 (0.025)	0.685 (0.018)	0.03 (0.023)	0.019 (0.018)
NUE	0.009 (0.009)	0.009 (0.009)	0.009 (0.008)	0.009 (0.008)	0.009 (0.008)	0.009 (0.008)	0.009 (0.009)	0.017 (0.014)	0.009 (0.009)	0.009 (0.009)	0.009 (0.009)	0.009 (0.008)	0.009 (0.009)	0.01 (0.009)	0.009 (0.009)	0.796 (0.029)	0.054 (0.024)
CUN	0.009 (0.009)	0.008 (0.008)	0.008 (0.007)	0.008 (0.007)	0.007 (0.007)	0.007 (0.007)	0.007 (0.007)	0.035 (0.025)	0.008 (0.008)	0.008 (0.008)	0.009 (0.008)	0.01 (0.009)	0.008 (0.008)	0.009 (0.009)	0.008 (0.007)	0.014 (0.013)	0.8325 (0.035)

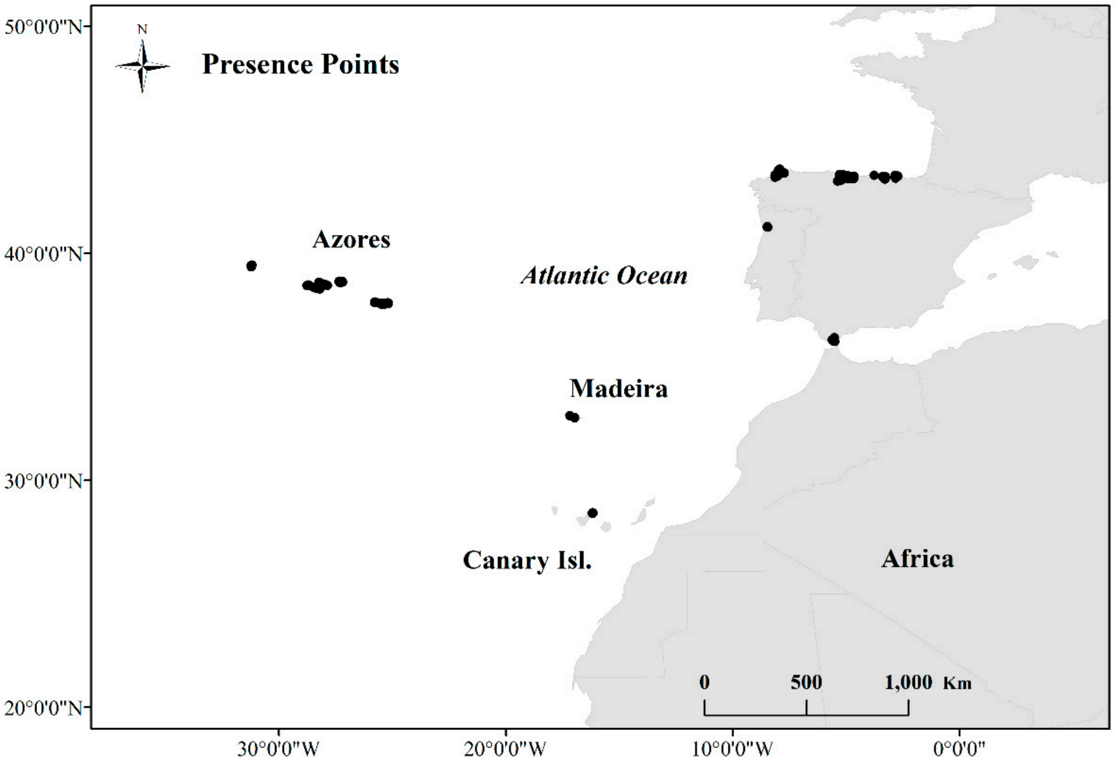

Figure S1. Location of presence records (black dots) used for species distribution modelling (SDM).

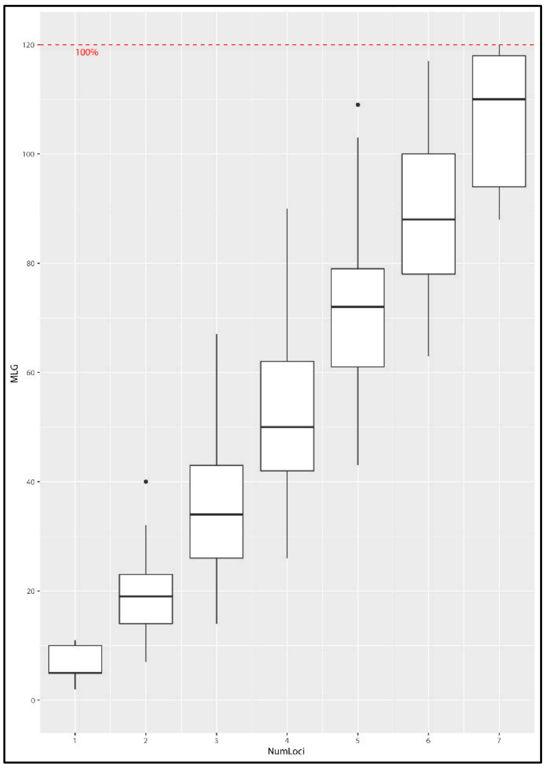

Figure S2. Genotypic accumulation curve showing the resolutive power of the eight microsatellite used in this study.

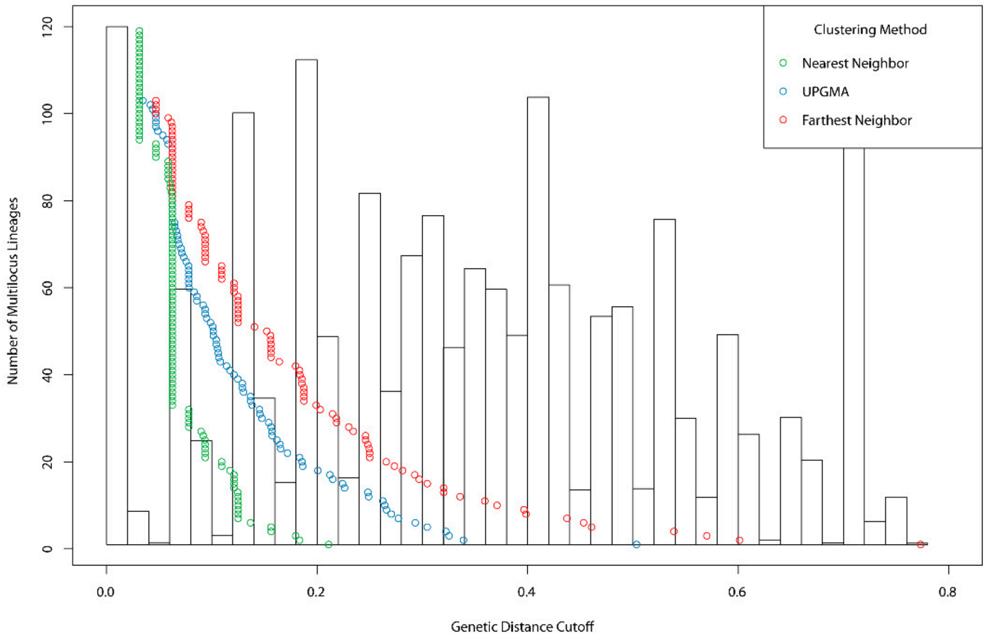

Figure S3. Histogram of frequency distribution of pairwise genetic distances. The genetic threshold distances (for the three methods implemented in POPPR) under which two multilocus genotypes are considered the same multilocus lineage are showed.

(A)

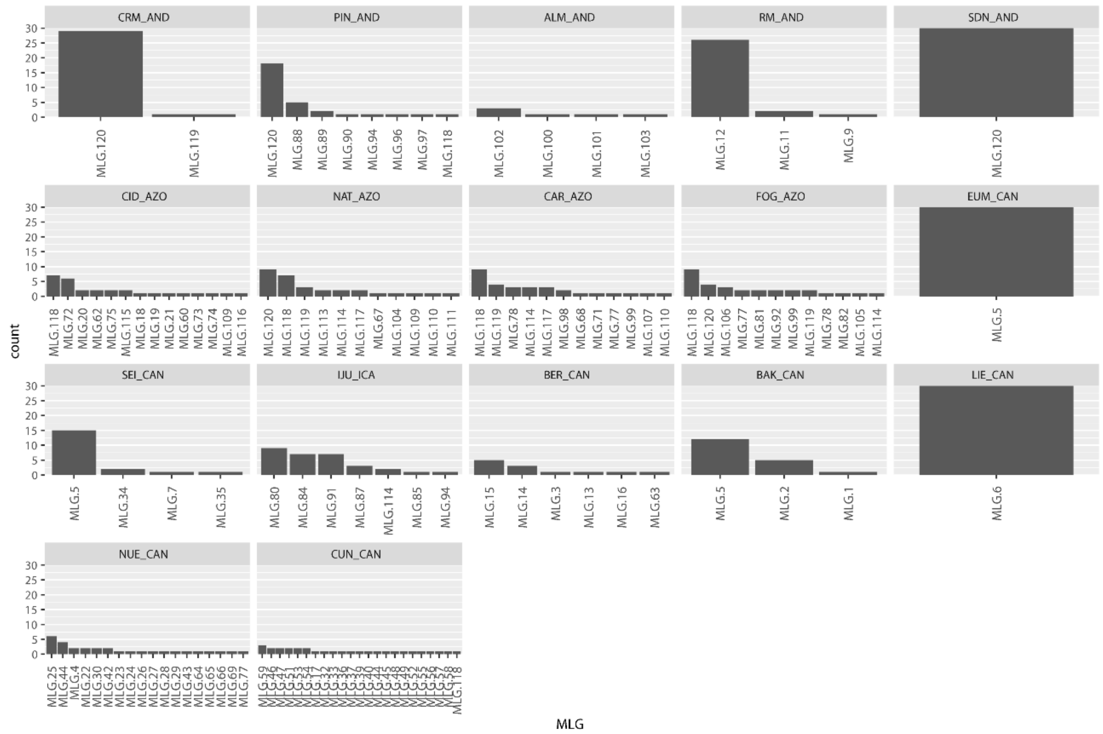

(B)

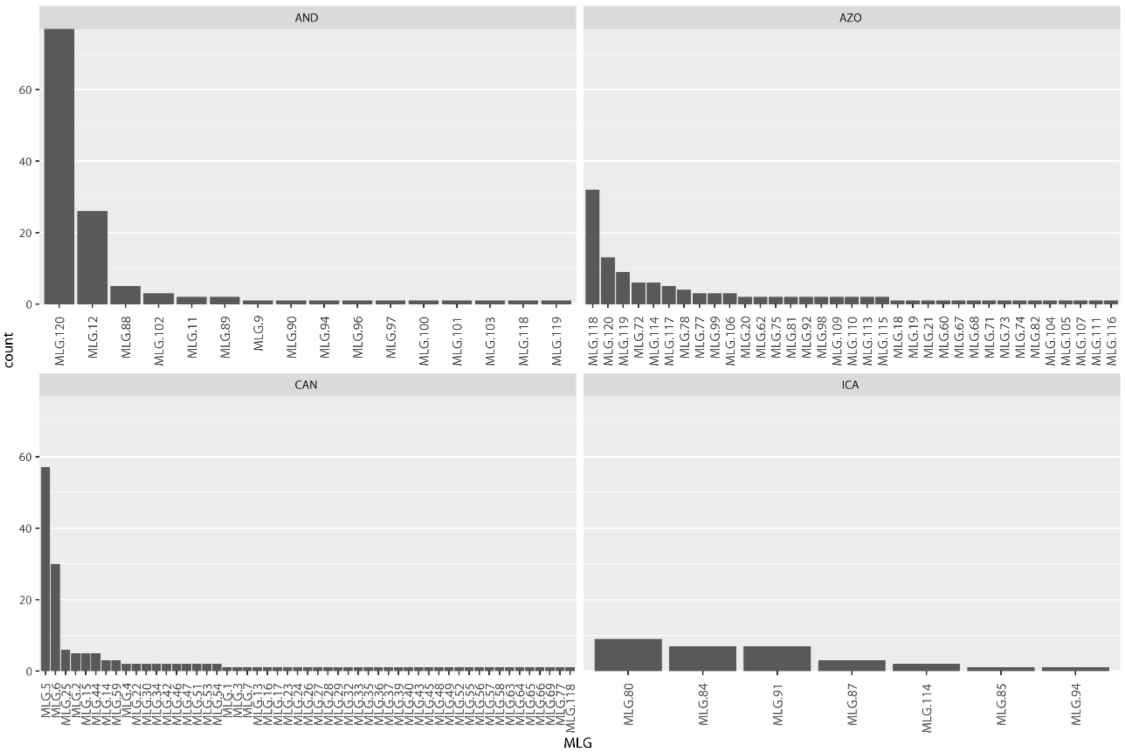

Figure S4. Distribution of the 104 multilocus lineages MLLs among the 130 individuals (genets) of *Culcita macrocarpa*. (**A**), Across the 17 populations. (**B**), Across the 4 geographical regions. For population codes, see Table 1.

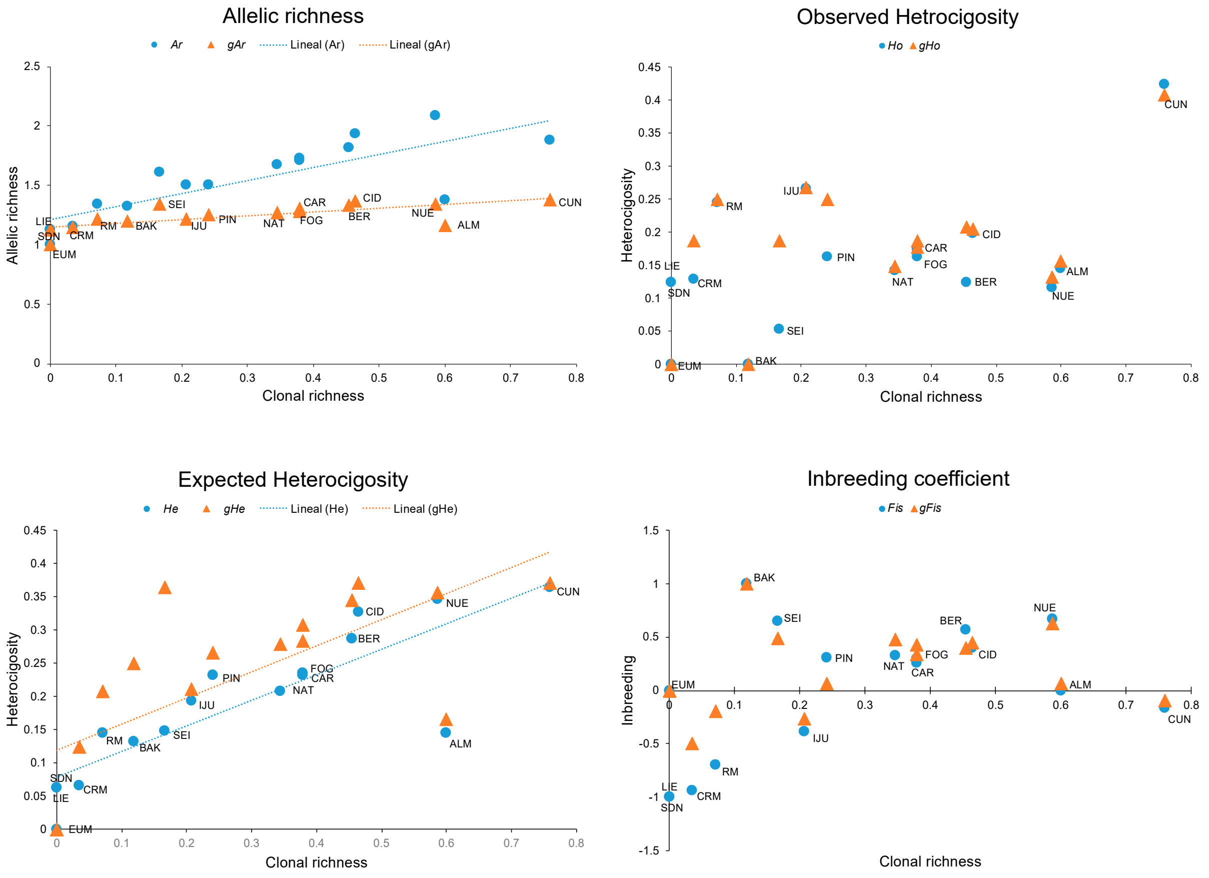

Figure S5. Scatterplots of genetic diversity estimates, obtained with eight microsatellite loci, against clonal richness (R) of *Culcita macrocarpa* populations. Indices calculated including all individuals sampled per population (blue circles) and including only one individual per multilocus lineage per population (*gIndex*; red triangles) are shown. For population codes, see Table 1.

Figure S6. Bar plots showing the STRUCTURE results, using microsatellite data and assuming the non admixture model. On the left, when all sampling units were considered; on the right, when only one individual per multilocus lineage per population was used. Delta *K* method [3] was used to identify the uppermost hierarchical level of genetic structure, while MedMedK, MedMeaK, MaxMedK, and MaxMeaK (with membership coefficient threshold of 0.5; [4] were used to identify other levels of genetic partitioning. The *K* values selected by each estimator are shown on the top. For population codes, see Table 1.

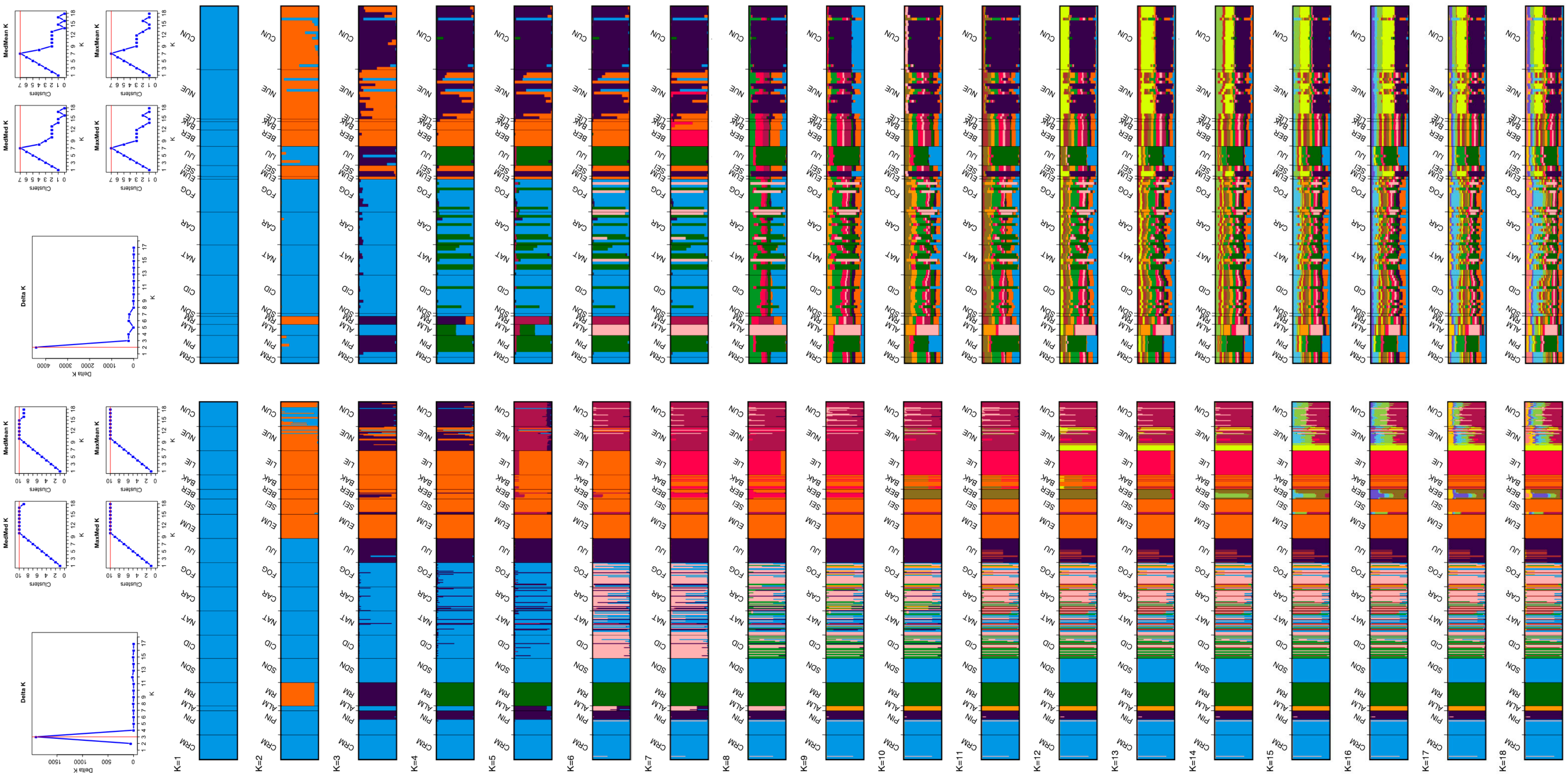

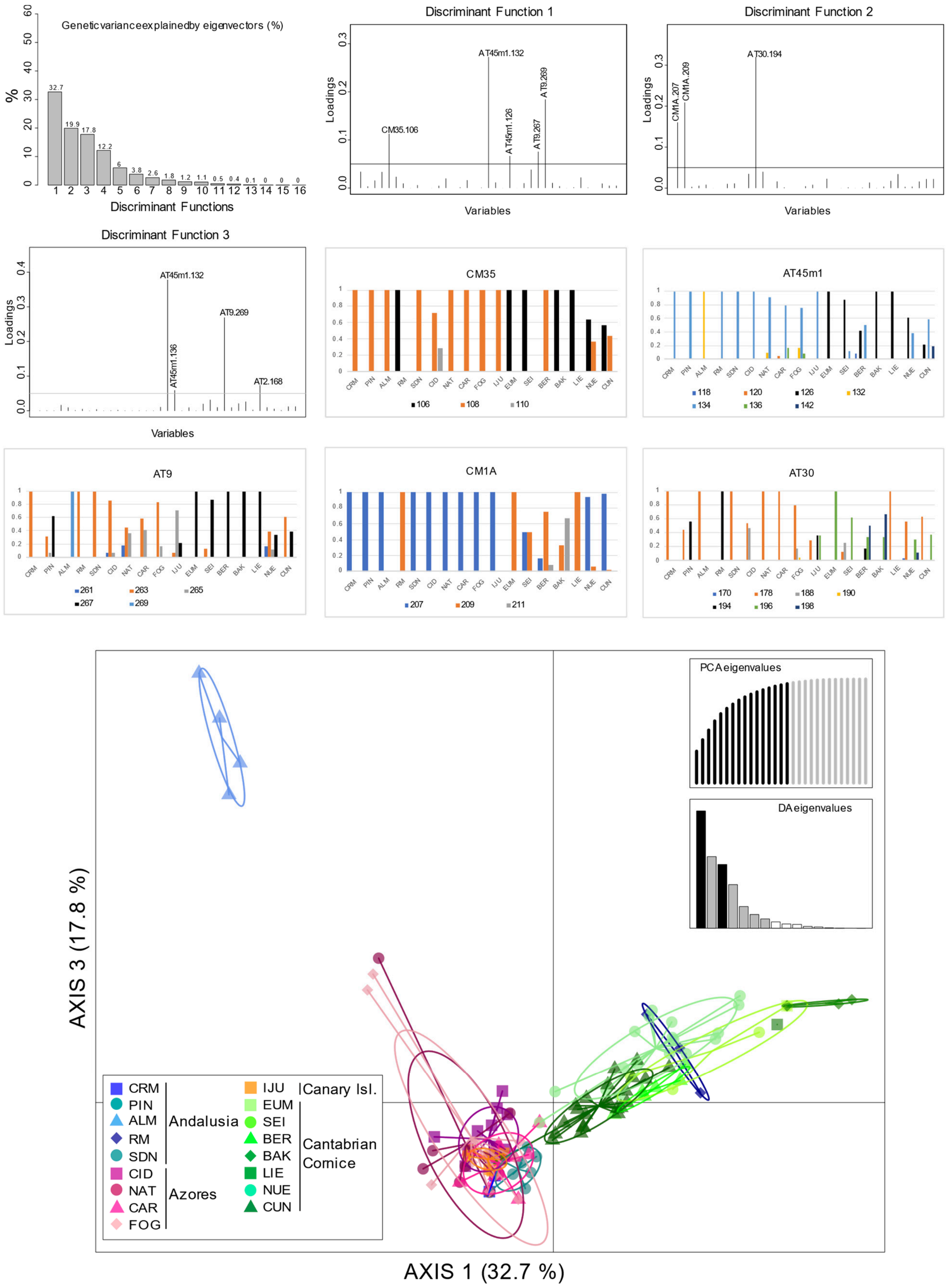

Figure S7. Additional results of the discriminant analysis of principal components (DAPC). Genetic variance explained by the discriminant functions, individual variable loadings for the first three discriminant functions, frequency distribution of the alleles with the highest resolution value for individual assignment, and scatterplot showing the first and third principal components of the DAPC.

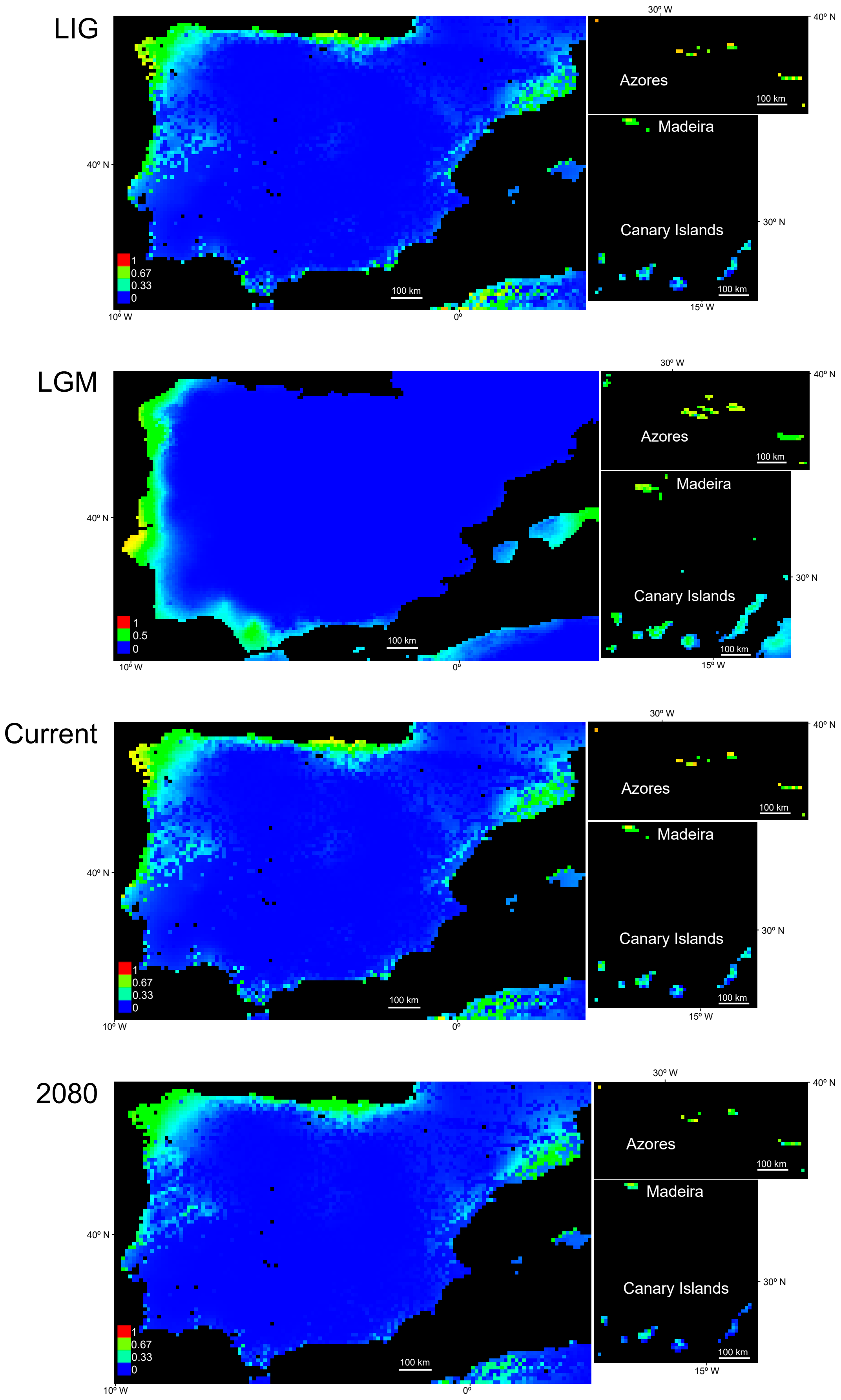

Figure S8. Detailed potential distribution of *Culcita macrocarpa* in the Iberian Peninsula and Macaronesian Islands drawn with MAXENT. The order of the time sequence is, from top to bottom: at the Last Interglacial (LIG, *ca.* 120,000 years BP); at the Last Glacial Maximum (LGM, *ca.* 21,000 years BP); at the present time (Current); at the Last Interglacial (LIG, *ca.* 120,000 years BP), using the Community Climate System Model (CCSM); prediction for the year 2080 under RCP 8.5 conditions.

References

1. Taberlet, P.; Gielly, L.; Pautou, G.; Bouvet, J. Universal primers for amplification of three non-coding regions of chloroplast DNA. *Plant Mol. Biol.* **1991**, *17*, 1105–1109, doi:10.1007/BF00037152.
2. Shaw, J.; Lickey, E.B.; Beck, J.T.; Farmer, S.B.; Liu, W.; Miller, J.; Siripun, K.C.; Winder, C.T.; Schilling, E.E.; Small, R.L. The tortoise and the hare II: Relative utility of 21 noncoding chloroplast DNA sequences for phylogenetic analysis. *Am. J. Bot.* **2005**, *92*, 142–166, doi:10.3732/ajb.92.1.142.
3. Evanno, G.; Regnaut, S.; Goudet, J. Detecting the number of clusters of individuals using the software STRUCTURE: A simulation study. *Mol. Ecol.* **2005**, *14*, 2611–2620, doi:10.1111/j.1365-294X.2005.02553.x.
4. Puechmaille, S.J. The program structure does not reliably recover the correct population structure when sampling is uneven: Subsampling and new estimators alleviate the problem. *Mol. Ecol. Resour.* **2016**, *16*, 608–627, doi:10.1111/1755-0998.12512.
